# Integration of longitudinal deep-radiomics and clinical data improves the prediction of durable benefits to anti-PD-1/PD-L1 immunotherapy in advanced NSCLC patients

**DOI:** 10.1186/s12967-023-04004-x

**Published:** 2023-03-05

**Authors:** Benito Farina, Ana Delia Ramos Guerra, David Bermejo-Peláez, Carmelo Palacios Miras, Andrés Alcazar Peral, Guillermo Gallardo Madueño, Jesús Corral Jaime, Anna Vilalta-Lacarra, Jaime Rubio Pérez, Arrate Muñoz-Barrutia, German R. Peces-Barba, Luis Seijo Maceiras, Ignacio Gil-Bazo, Manuel Dómine Gómez, María J. Ledesma-Carbayo

**Affiliations:** 1 Biomedical Image Technologies, ETSI Telecomunicación, Universidad Politécnica de Madrid, 28040 Madrid, Spain; 2 Centro de Investigación Biomédica en Red de Bioingeniería, Biomateriales y Nanomedicina (CIBER-BBN), Madrid, Spain; 3 Hospital Universitario Fundación Jiménez Díaz, 28040 Madrid, Spain; 4 Clínica Universidad de Navarra, 28027 Madrid, Spain; 5 Centro de Investigación Biomédica en Red de Enfermedades Respiratorias (CIBERES), Pamplona, Spain; 6 Centro de Investigación Biomédica en Red de Cáncer (CIBERONC), 31008 Pamplona, Spain; 7 Bioengineering Department, Universidad Carlos III de Madrid, 28911 Leganés, Spain; 8 Instituto de Investigación Sanitaria Gregorio Marañón, 28007 Madrid, Spain; 9 Department of Oncology, Clínica Universidad de Navarra, 31008 Pamplona, Spain; 10 Program in Solid Tumors, Center for Applied Medical Research (CIMA), 31008 Pamplona, Spain; 11 Navarra Institute for Health Research, IdiSNA, 31008 Pamplona, Spain; 12 Department of Oncology, Fundación Instituto Valenciano de Oncología (FIVO), 46009 Valencia, Spain

**Keywords:** Immunotherapy, Lung cancer, Clinical durable benefit, Deep-Radiomics, Clinical data, Longitudinal analysis, Treatment monitoring

## Abstract

**Background:**

Identifying predictive non-invasive biomarkers of immunotherapy response is crucial to avoid premature treatment interruptions or ineffective prolongation. Our aim was to develop a non-invasive biomarker for predicting immunotherapy clinical durable benefit, based on the integration of radiomics and clinical data monitored through early anti-PD-1/PD-L1 monoclonal antibodies treatment in patients with advanced non-small cell lung cancer (NSCLC).

**Methods:**

In this study, 264 patients with pathologically confirmed stage IV NSCLC treated with immunotherapy were retrospectively collected from two institutions. The cohort was randomly divided into a training (n = 221) and an independent test set (n = 43), ensuring the balanced availability of baseline and follow-up data for each patient. Clinical data corresponding to the start of treatment was retrieved from electronic patient records, and blood test variables after the first and third cycles of immunotherapy were also collected. Additionally, traditional radiomics and deep-radiomics features were extracted from the primary tumors of the computed tomography (CT) scans before treatment and during patient follow-up. Random Forest was used to implementing baseline and longitudinal models using clinical and radiomics data separately, and then an ensemble model was built integrating both sources of information.

**Results:**

The integration of longitudinal clinical and deep-radiomics data significantly improved clinical durable benefit prediction at 6 and 9 months after treatment in the independent test set, achieving an area under the receiver operating characteristic curve of 0.824 (95% CI: [0.658,0.953]) and 0.753 (95% CI: [0.549,0.931]). The Kaplan-Meier survival analysis showed that, for both endpoints, the signatures significantly stratified high- and low-risk patients (p-value< 0.05) and were significantly correlated with progression-free survival (PFS6 model: C-index 0.723, p-value = 0.004; PFS9 model: C-index 0.685, p-value = 0.030) and overall survival (PFS6 models: C-index 0.768, p-value = 0.002; PFS9 model: C-index 0.736, p-value = 0.023).

**Conclusions:**

Integrating multidimensional and longitudinal data improved clinical durable benefit prediction to immunotherapy treatment of advanced non-small cell lung cancer patients. The selection of effective treatment and the appropriate evaluation of clinical benefit are important for better managing cancer patients with prolonged survival and preserving quality of life.

**Supplementary Information:**

The online version contains supplementary material available at 10.1186/s12967-023-04004-x.

## Introduction

Immunotherapy has radically changed the therapeutic paradigm in cancer, becoming the new standard for treating locally advanced and metastatic non-small cell lung cancer (NSCLC) patients [1]. Many studies have shown positive results in terms of improved long-term survival when used alone or in combination with other treatments [2–5], but only a small proportion of patients (20–50%) respond to therapy [6, 7]. Due to immunotherapy’s unconventional response pattern, including delayed response or pseudoprogression, traditional approaches to defining response are no longer adequate. Furthermore, patients may experience immune-related adverse events, which can be life threatening [8].

It has become crucial to identify biomarkers that could predict long-term clinical benefit patients to monitor their condition over time effectively. Different biomarkers have been investigated, such as PD-L1 expression and tumor mutational burden, and their association with treatment response has been reported in previous studies with mixed results [9, 10]. Furthermore, tumor heterogeneity could influence the reliability of these biomarkers, as they depend on biopsied tissue, which cannot cover the entire tumor microenvironment.

The use of non-invasive image-based biomarkers has gained increased attention during the past few years because of their availability and non-invasiveness. Typically, the effectiveness of treatment has been evaluated using the response evaluation criteria in solid tumors (RECIST) [11] or its adaptation to immunotherapy (iRECIST) [12]. However, these criteria are often subjective and do not consider changes in tumor heterogeneity.

Radiomics involves the high-throughput extraction of a large number of quantitative characteristics from medical imaging, which can provide complete information on tumor radiophenotype and microenvironment heterogeneity [13]. Several studies have demonstrated the ability of radiomics features to predict the immunotherapy response for advanced NSCLC patients, uncovering characteristics that otherwise could not be identified by human observers [14–18]. In addition, recent advances in deep learning have shown that radiomics features can be automatically extracted using neural networks without human feature interaction, resulting in better prediction performance (deep-radiomics) [19, 20]. Most of these studies have focused on the development of biomarkers considering only baseline and first follow-up information. However, given that tumors are heterogeneous in terms of both spatial heterogeneity and temporal evolution, it could be beneficial to consider more temporal information during early treatment to understand better tumor response patterns.

Furthermore, integrating multimodal data, such as clinical and imaging data, could provide complementary patient- and tumor-specific information for better patient monitoring [21].

The present study aimed to investigate the potential improvements in prediction performance by integrating imaging and clinical data monitored through early treatment. The ability of deep learning to extract more complex and response-related features was also explored and compared with traditional radiomics. An ensemble model based on the integration of longitudinal radiomics and clinical data has been developed and validated in an independent test set to predict the clinical durable benefit of immunotherapy in patients with NSCLC at 6 and 9 months after the start of treatment.

## Materials and methods

### Datasets and patient selection

A total of 291 patients with pathologically confirmed stage IV NSCLC treated with anti-PD-1/PD-L1 monoclonal antibodies from January 2013 to December 2021 were retrospectively collected at the Hospital Universitario Fundación Jiménez Díaz (FJD, 154 patients) and Clínica Universidad de Navarra (CUN, 137 patients). Their institutional review boards approved the study, and informed consent was collected accordingly. Inclusion criteria were: (a) confirmed advanced NSCLC; (b) patients were treated with immunotherapy as monotherapy, a combination of immuno-based agents, or in combination with traditional treatment such as chemotherapy or radiation therapy; (c) availability of clinical and epidemiological information; (d) patient data were not right-censored. Finally, 264 patients were enrolled in this study.

The institutional medical records systems were searched to identify those patients with imaging data. CT images were available for 186 patients and were collected following these inclusion criteria: (a) availability of chest CT scans; (b) availability of baseline CT within 2 months before the start of immunotherapy. Exclusion criteria were as follows: (a) lung resection during treatment; (b) an experienced radiologist could not detect and segment the primary tumor in the baseline CT; (c) poor quality image; (d) patient data were not right-censored. Finally, 171 patients were enrolled for imaging data analysis.

According to the clinical protocol, during the first 4 months of immunotherapy, CT scans were acquired after every two or three treatment cycles. Conventional clinical evaluations (including hemograms) were performed after each treatment cycle within the first 2 months of treatment. As a result, our data included demographic, epidemiological, hemogram and other conventional clinical data from this period, at least a baseline CT scan and up to two follow-up CT scans.

**Fig. 1:**
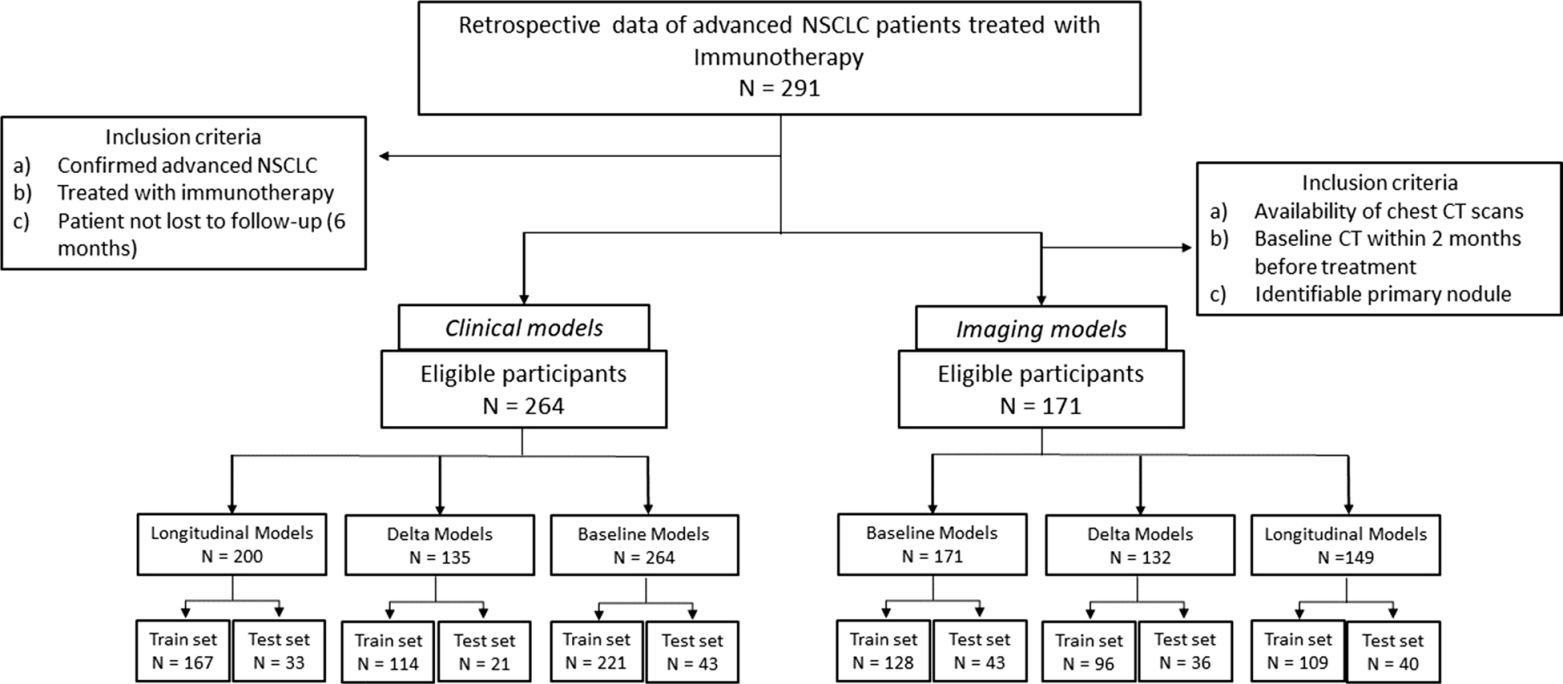
Flowchart showing the inclusion and exclusion criteria considering the endpoint PFS6. Details of the number of patients in the training and independent test set are provided

The cohort was divided into a training set and an independent test set balancing the availability of baseline and follow-up data (Fig. 1). To compare the performance of the different models, 43 patients (43/171 = 25%) with baseline imaging and clinical data were randomly selected as an independent test set to maximize the number of patients available to test all the implemented models. All remaining patients were used as the discovery set (n = 221). Among the independent test cohort patients, 40 had longitudinal imaging data, 33 longitudinal clinical data and 32 had both longitudinal imaging and clinical data (see details in Additional file 1: Tables S1 and S2).

### Clinical endpoints

The primary endpoint of this study was the durable clinical benefit defined by progression-free survival (PFS). It measures the time from the first cycle of immunotherapy to death/disease progression or last follow-up. Disease progression was defined based on the patient’s general clinical status and iRECIST criteria derived from the imaging evaluation. Patients with durable clinical benefit that had a PFS longer than 6 (PFS6) or 9 (PFS9) months were denominated as responders, while the others as nonresponders [22]. Patients with censored data 6 or 9 months after treatment were excluded from the analysis. The maximum follow-up period was 48 months.

The secondary endpoint was overall survival (OS), defined as the time in months between the initiation of immunotherapy and death or censored to the last follow-up visit for survivors.

### Image acquisition and pre-processing

All patients underwent a CT scan within 2 months before the immunotherapy treatment start date. When available, follow-up CT scans were acquired within 4 months after treatment (up to three temporal time points per patient). All CT images were acquired after contrast injection during a patient inspiratory breath hold, following the contrast-enhanced CT chest protocol. CT scans were reconstructed using a standard kernel. A description of CT parameters is available in Additional file 1: Table S3.

For each case, the primary tumor was selected as the target lesion. 3D tumors were identified and segmented by an experienced radiologist on the baseline and follow-up CT images using either the syngo.via Siemens Healthineers software or 3D Slicer [23]. The largest lesion was considered if a patient had an ambiguous primary tumor. Follow-up CT scans were discarded if the tumor found in the baseline CT scan was no longer visible.

For pre-processing, Hounsfield units of all CT images were clipped between -1000 and 3050, and z-score normalization was then applied.

### Feature engineering

#### Radiomics analysis

Radiomics features were extracted by using Pyradiomics (version 3.0.1) [24]. The voxel intensity values were discretized when computing some texture features using a bin width of 25 Hounsfield units [25]. To reduce the effect of low resolution along the z-axis in part of the data, the radiomics features were computed only by applying 2D filters.

Feature reproducibility and feature repeatability against segmentation were assessed using the QIN Lung CT Segmentation dataset, a random subset of the data, and the RIDER dataset (Additional file 1: S3). Reproducible and repeatable features are potentially more robust to variations in CT scanners, acquisition parameters, and segmentation.

After feature extraction and reproducibility selection, delta-radiomics features were calculated as the relative net change between features at baseline and first follow-up CTs. Patients without first follow-up CT were discarded from this analysis.

A standard scaler was applied to normalize each radiomics feature. The transformation was learned in training and then applied to the test set.

#### Deep feature extraction

To extract high-level and domain-related representations (e.g., texture, morphology) of the tumors’ deep learning-based features, the convolutional neural network (CNN) architecture NoduleX [26] was used as a reference implementation to predict the response to immunotherapy. NoduleX input consists of a small 3D volume of $47\times 47$ pixels $\times$ 5 slices centered in the centroid of the tumor that was sampled and resized from a square of $10\times 10$ cm$^{2}$. Image intensities were clipped to the range [-1000, 3050] and then normalized.

A transfer learning approach was used to pretrain NoduleX CNN architecture weights. Namely, the network was pre-trained to predict the malignancy of tumors collected from 719 patients of The Lung Image Database Consortium and Image Database Resource Initiation Data Set (LIDC-IDRI) [27] and 14 patients who did not meet the inclusion criteria of the immunotherapy dataset (1528 tumors, Additional file 1: S4). Then, the last two convolutional layers and the classification layers were fine-tuned to predict the response (defined by the endpoint PFS6). For network fine-tuning, all primary tumors of all available CT images from the immunotherapy training data set (357 tumors - 128 patients) were used. Fine-tuning allowed the efficient transfer of malignant-related spatial features to more complicated high-level semantic features related to immunotherapy response.

After training, deep features were extracted for each tumor from the first fully connected layers of the network (500 deep features), referred to as DF-imm. Similarly to delta-radiomics, delta DF-imm features were also calculated.

### Clinical data

Baseline demographic, epidemiological, clinical and laboratory data were collected from electronic patient records, as well as hemogram-related data after the second and third treatment cycles. They included sex, age, body mass index, tumor histology, smoking, previous surgery, presence of metastases, and immune cell-related indexes, among others (Additional file 1: S5).

One hot encoding was applied to categorical or constant variables. Z-score normalization was applied to continuous variables, and missing data were imputed using the k-means algorithm. Delta features were also calculated.

### Model design and analysis

Random Forest (RF) models were built for each primary endpoint in the training set using stratified three-fold cross-validation. The number of training patients for each RF model is reported in Additional file 1:Tables S1 (PFS6) and S2 (PFS9). Feature selection and RF hyperparameter optimization were performed using a Bayesian optimization approach. The optimized hyperparameters were the number of estimators, the maximum depth, and the number of features.

Radiomics, deep features, and clinical data were used to implement baseline, delta, and longitudinal RF models trained for predicting the immunotherapy response. Baseline models’ (RF-baseline) inputs were only the data before the start of treatment, whereas longitudinal models used baseline and early treatment data. Patients who did not have follow-up data were excluded from the longitudinal analysis. Two types of longitudinal models were constructed: RF-delta and RF-longitudinal. RF-delta model had delta features as input and considered only patients with baseline and first follow-up data. On the other hand, RF-longitudinal input was the concatenation of all available features over time for each patient (number of features multiplied by the number of time points). Missing time points were imputed as the closest in time available data.

For comparison, the NoduleX architecture pre-trained for malignancy prediction was fine-tuned with the baseline training data of the immunotherapy dataset to predict treatment durable response (CNN-baseline).

For predicting PFS9, because the training was imbalanced, a synthetic minority oversampling technique (SMOTE) was used during the training phase to resample the minority class (“responders”). As SMOTE was configured to generate synthetic samples in training considering five nearest neighbors, the numbers of responders and nonresponders were equal.

**Fig. 2:**
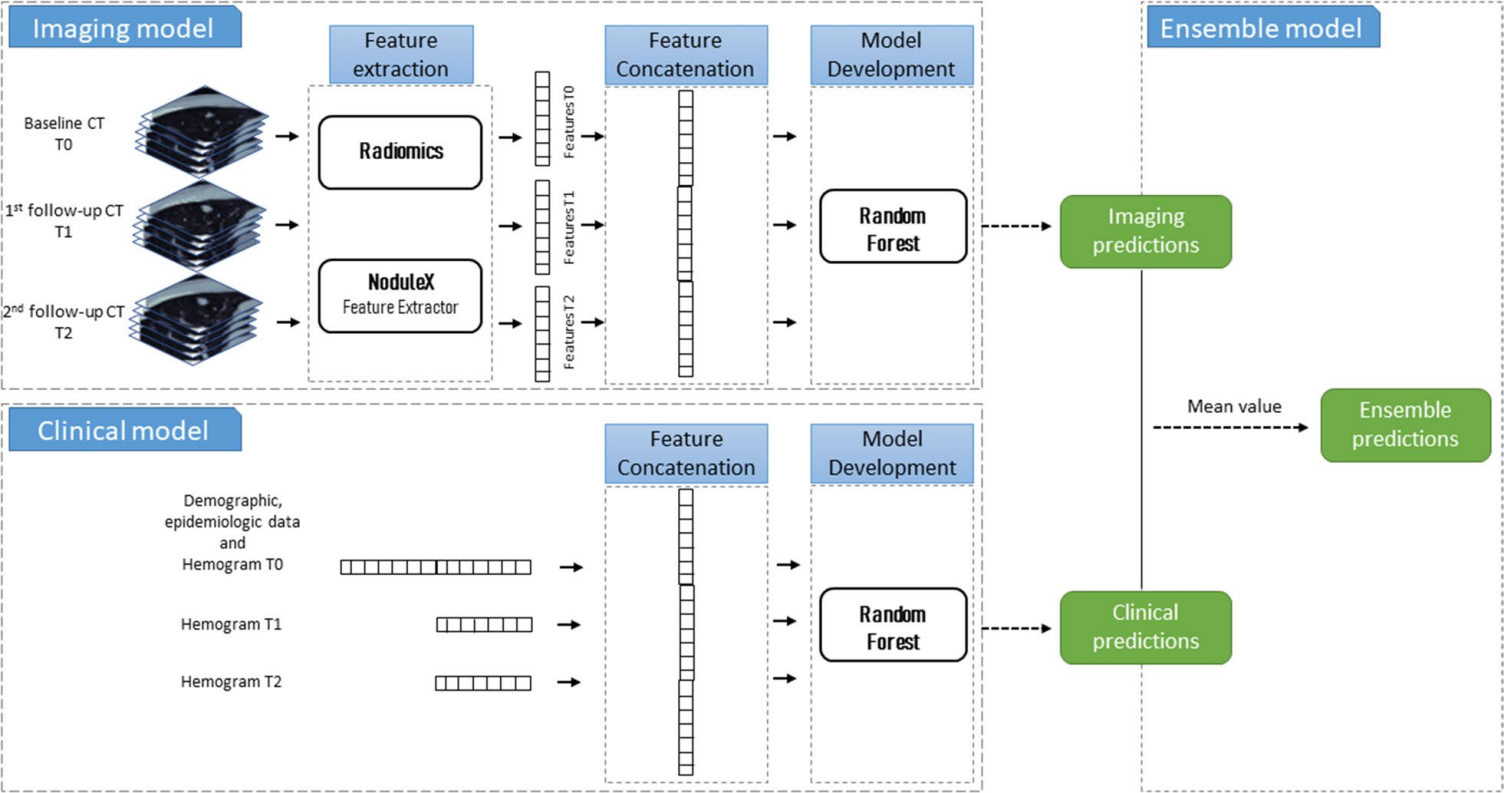
Implementation workflow of the longitudinal and ensemble models

Once the models were trained, ensemble RF models were implemented as the mean value of the predictions of the imaging and clinical models alone (ensemble RF). They allowed integrating both clinical and image information. The workflow is shown in Fig. 2.

### Model interpretation

The SHAP (or SHapley Additive exPlanations) algorithm was employed to visualize each feature’s contribution to producing the final prediction of the model [28]. SHAP assigns an importance value to each feature for each individual predicted value based on concepts from Cooperative Game Theory and local explanations. We applied the SHAP algorithm to the clinical model of the ensemble RF model. SHAP values were calculated to understand how much each feature impacted the model output or how much it increased or decreased the probability of a single outcome. SHAP values allowed us to determine whether the relationship between a feature and the output was correlative or anticorrelative. SHAP analysis was performed in Python using the KernelExplainer in the SHAP module (version 0.40.0).

### Statistical and survival analysis

Stratified three-fold cross-validation was performed in the training set to train all the implemented models and optimize the RF hyperparameters. Model performance was evaluated by the area under the receiver operating characteristic (ROC) curve (AUC) and the corresponding 95% confidence interval (CI) was estimated with a bootstrap resampling approach (1000 iterations). The differences between ROC curves were assessed using the DeLong test. Kaplan-Meier survival analysis was performed for patients’ stratification based on the model’s predictions (threshold = 0.5). The significance of differences between survival curves was assessed with the log-rank test. Hazard ratios (HRs) and concordance index were calculated using the Cox proportional-hazards model. p-values less than 0.05 (two-sided tests) were considered significant. R (version 4.1.1) and Python (version 3.7.10) were used for statistical analysis and model implementation.

## Results

### Patient characteristics

The clinical characteristics of patients in the training and independent test cohorts in the baseline and longitudinal analysis for PFS6 are summarized in Table 1. The characteristics of a subset of patients with imaging data are summarized in Additional file 1: S6 and S7. The same distributions were verified for PFS9.

**Table 1:** Demographic and clinical characteristics of the patients in the baseline and longitudinal analyses. P-values of no significant difference analysis (p-value> 0.05) between the training and test set after two samples T-test for continuous variables, and Chi-square test for categorical variables. SD represents the standard deviation, and Q1 and Q3 represent the first and third quartiles, respectively

Characteristic	Baseline analysis				Longitudinal analysis			
	All patients (N= 264)	Train set (N = 221)	Test set (N = 43)	P-value	All patients (N= 200)	Train set (N = 167)	Test set (N = 33)	p-value
PFS, mean (SD)	9.0 (11.1)	9.3 (11.6)	7.6 (8.1)	0.242	11.1 (11.8)	11.6 (12.3)	9.0 (8.6)	0.147
OS, mean (SD)	13.3 (12.2)	13.3 (12.5)	13.5 (10.5)	0.903	16.0 (12.4)	16.0 (12.8)	15.7 (10.6)	0.889
Status								
Alive	107 (40.5%)	91 (41.2%)	16 (37.2%)	0.753	91 (45.5)	78 (46.7)	13 (39.4)	0.562
Dead	157 (59.5%)	130 (58.8%)	27 (62.8%)		109 (54.5)	89 (53.3)	20 (60.6)	
Response								
Non-responders	148 (56.1%)	124 (56.1%)	24 (55.8%)	1.000	90 (45.0%)	75 (44.9%)	15 (45.5%)	1.000
Responders	116 (43.9%)	97 (43.9%)	19 (44.2%)		110 (55.0%)	92 (55.1%)	18 (54.5%)	
Progression								
No progression	45 (17.0%)	40 (18.1%)	5 (11.6%)	0.417	42 (21.0%)	38 (22.8%)	4 (12.1%)	0.256
Progression	219 (83.0%)	181 (81.9%)	38 (88.4%)		158 (79.0%)	129 (77.2%)	29 (87.9%)	
Age, median [Q1,Q3]	65.0 [59.0,71.0]	65.0 [58.0,71.0]	67.0 [60.5,72.5]	0.204	65.0 [58.0,70.2]	64.0 [57.0,70.0]	67.0 [60.0,72.0]	0.266
Sex								
Female	80 (30.3%)	66 (29.9%)	14 (32.6%)	0.865	58 (29.0%)	47 (28.1%)	11 (33.3%)	0.696
Male	184 (69.7%)	155 (70.1%)	29 (67.4%)		142 (71.0%)	120 (71.9%)	22 (66.7%)	
IPA, mean (SD)	45.2 (33.4)	45.1 (33.8)	45.4 (31.5)	0.958	44.0 (34.1)	44.9 (34.6)	39.0 (31.2)	0.357
Smoking								
Current smoker	55 (21.0%)	50 (22.7%)	5 (11.9%)	0.258	39 (19.7%)	35 (21.1%)	4 (12.5%)	0.530
Former smoker	180 (68.7%)	147 (66.8%)	33 (78.6%)		135 (68.2%)	111 (66.9%)	24 (75.0%)	
Non-smoker	27 (10.3%)	23 (10.5%)	4 (9.5%)		24 (12.1%)	20 (12.0%)	4 (12.5%)	
Tumour histology								
Adenocarcinoma	203 (76.9%)	170 (76.9%)	33 (76.7%)	0.897	151 (75.5%)	126 (75.4%)	25 (75.8%)	0.896
Epidermoid carcinoma	52 (19.7%)	43 (19.5%)	9 (20.9%)		40 (20.0%)	33 (19.8%)	7 (21.2%)	
Other	9 (3.4%)	8 (3.6%)	1 (2.3%)		9 (4.5%)	8 (4.8%)	1 (3.0%)	
PDL1, mean (SD)	0.4 (0.4)	0.4 (0.4)	0.4 (0.4)	0.876	0.4 (0.4)	0.4 (0.4)	0.3 (0.3)	0.194
Surgery								
No	227 (86.0%)	190 (86.0%)	37 (86.0%)	1.000	171 (85.5%)	142 (85.0%)	29 (87.9%)	0.792
Yes	37 (14.0%)	31 (14.0%)	6 (14.0%)		29 (14.5%)	25 (15.0%)	4 (12.1%)	
Treatment								
Combined immunological agents	39 (14.8%)	29 (13.1%)	10 (23.3%)	0.393	31 (15.5%)	24 (14.4%)	7 (21.2%)	0.276
Immunotherapy + chemotherapy	50 (18.9%)	41 (18.6%)	9 (20.9%)		39 (19.5%)	30 (18.0%)	9 (27.3%)	
Immunotherapy + radiotherapy	17 (6.4%)	15 (6.8%)	2 (4.7%)		11 (5.5%)	11 (6.6%)	0 (0%)	
Monotherapy	154 (58.3%)	132 (59.7%)	22 (51.2%)		116 (58.0%)	99 (59.3%)	17 (51.5%)	
Other	4 (1.5%)	4 (1.8%)	0 (0%)		3 (1.5%)	3 (1.8%)	0 (0%)	

Among the selected 264 patients, 80 were female (mean age, 62.6 ± 9.8 [standard deviation]) and 184 were male (mean age, 65.7 ± 9.7 [standard deviation]). Regarding our cohort, we found the following: 43.9% of the patients responded to immunotherapy after 6 months of treatment, while only 33.2% responded after 9 months; adenocarcinoma was the most prevalent histological variant of advanced NSCLC (76.9%); and 89.7% of the patients were current or former smokers. Immunotherapy treatment included monotherapy (58.3%), immunotherapy combined with radiation therapy (6.4%), immunotherapy combined with chemotherapy (18.9%) and a combination of different immunological agents (14.8%). No demographic or clinical characteristics had significant differences (p-value < 0.05) between the training and test set after the two samples of T-tests for continuous variables and Chi-square tests for categorical variables.

For the subcohort of patients with imaging data (171 over 264 patients), the training and the independent test sets had identical distributions of demographics and clinical characteristics (no statistical difference p > 0.05).

### Model development and response prediction performance

From the initial set of 1365 radiomics features, only 173 (13%) verified both reproducibility and repeatability against segmentation tests. Furthermore, a total of 500 DF-imm were extracted for each tumor using the NoduleX architecture. The number of features used as input varied depending on each model. The number of features selected for each implemented model and the results in the training set are shown in Additional file 1: Tables S8 and S9, respectively.

**Fig. 3:**
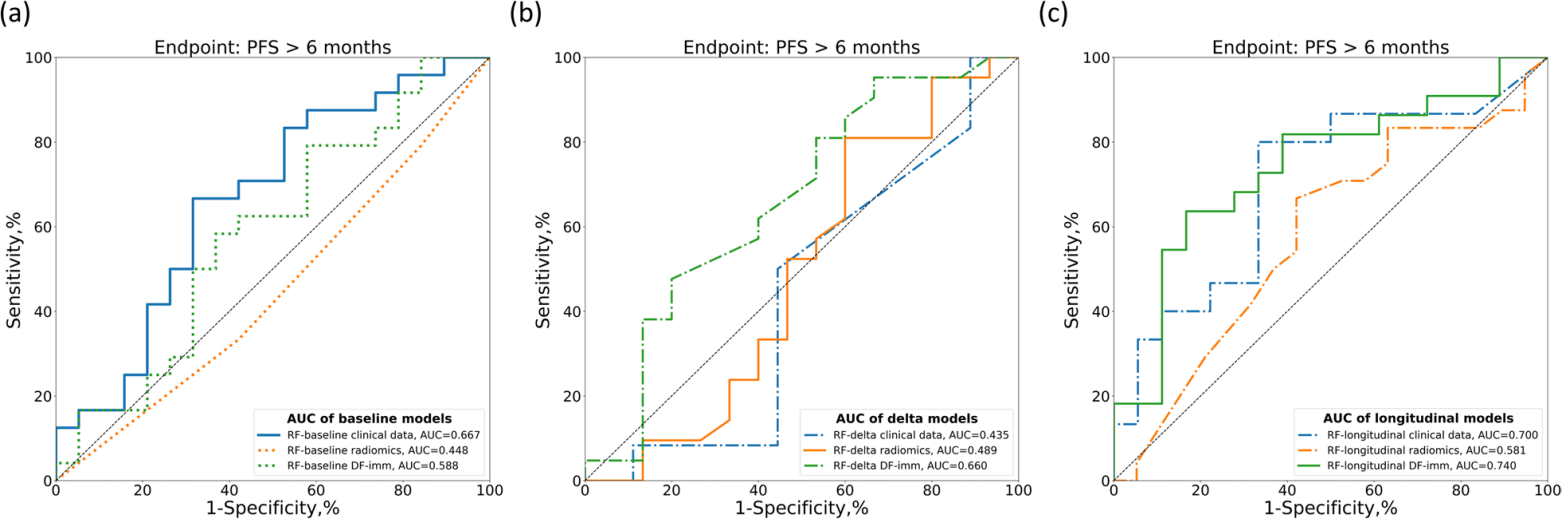
Comparisons of the ROC curves for endpoint PFS6 prediction of response of the baseline (**a**), delta (**b**), and longitudinal RF models (**c**) based on clinical, radiomics, or deep-radiomics data

**Fig. 4:**
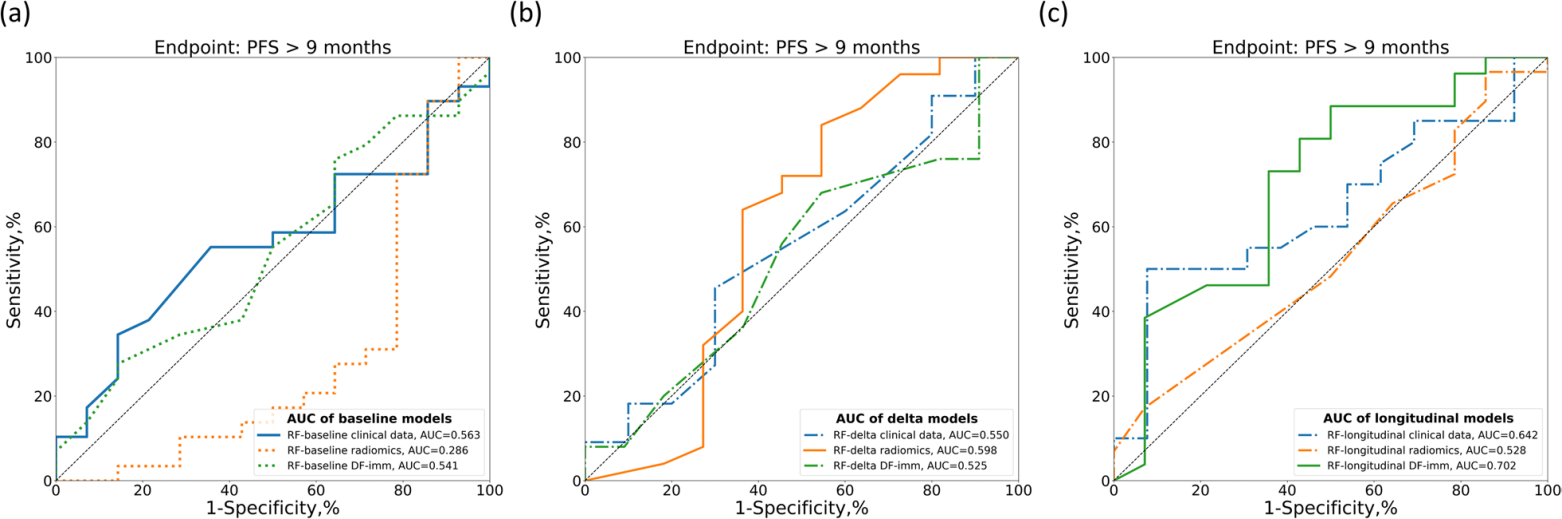
Comparisons of the ROC curves for endpoint PFS9 prediction of response of the baseline (**a**), delta (**b**), and longitudinal RF models (**c**) based on clinical, radiomics, or deep-radiomics data

Figures 3 and 4 compare the ROC curves of CNN-baseline and the baseline, delta and longitudinal RF models using clinical, radiomics and DF-imm data in the independent test cohort for PFS6 and PFS9, respectively.

Longitudinal models performed better than baseline or delta models in the independent test cohort, achieving an AUC of 0.740 (95% CI: 0.563$-$0.833) with DF-imm and an AUC of 0.700 (95% CI: 0.508$-$0.877) with clinical data for PFS6 and an AUC of 0.702 (95% CI: 0.515$-$0.867) with DF-imm and an AUC of 0.585 (95% CI: 0.367$-$0.783) with clinical data for PFS9. In both cases, the automatically extracted features performed better than the hand-crafted radiomics features and clinical data (Figs. 3 and 4).

Tables 2 and 3 compare the evaluation metrics of all implemented models, showing great improvement when using the longitudinal models.

**Table 2:** Response prediction performance comparison between baseline, delta and longitudinal models in the independent test set for endpoint PFS6 by evaluating AUC, ACC, SENS, SPEC, PREC and bACC, respectively

Model	Features	N test	AUC [95% CI]	ACC [95% CI]	SENS [95% CI]	SPES [95% CI]	PREC [95% CI]	bACC [95% CI]
CNN-baseline	Image data	43	0.518 [0.329,0.696]	0.535 [0.372,0.674]	0.750 [0.565,0.909]	0.263 [0.067,0.478]	0.562 [0.387,0.737]	0.507 [0.377,0.643]
RF-baseline	Clinical data	43	0.667 [0.485,0.833]	0.651 [0.512,0.791]	**0.833** [0.667,0.962]	0.421 [0.200,0.650]	0.645 [0.480,0.812]	0.627 [0.488,0.774]
RF-baseline	Radiomics	43	0.448 [0.291,0.607]	0.442 [0.302,0.605]	0.333 [0.150,0.526]	0.579 [0.350,0.800]	0.500 [0.250,0.750]	0.456 [0.306,0.601]
RF-baseline	DF-imm	43	0.588 [0.409,0.767]	0.558 [0.419,0.698]	**0.833** [0.679,0.960]	0.211 [0.050,0.417]	0.571 [0.406,0.735]	0.522 [0.403,0.638]
RF-delta	Clinical data	21	0.435 [0.173,0.714]	0.333 [0.143,0.571]	0.167 [0.000,0.417]	0.556 [0.200,0.875]	0.333 [0.000,0.750]	0.361 [0.163,0.559]
RF-delta	Radiomics	36	0.489 [0.276,0.706]	0.528 [0.361,0.694]	0.524 [0.304,0.737]	0.533 [0.273,0.786]	0.611 [0.389,0.833]	0.529 [0.357,0.688]
RF-delta	DF-imm	36	0.660 [0.451,0.846]	0.611 [0.444,0.778]	0.714 [0.500,0.900]	0.467 [0.200,0.733]	0.652 [0.455,0.842]	0.590 [0.433,0.750]
RF-longitudinal	Clinical data	33	0.700 [0.508,0.877]	0.576 [0.394,0.727]	0.467 [0.200,0.733]	**0.667** [0.438,0.875]	0.530 [0.250,0.800]	0.567 [0.405,0.733]
RF-longitudinal	Radiomics	40	0.581 [0.407,0.749]	0.628 [0.488,0.767]	0.667 [0.464,0.850]	0.579 [0.348,0.800]	0.667 [0.474,0.852]	0.623 [0.466,0.763]
RF-longitudinal	DF-imm	40	**0.740** [0.563,0.883]	**0.700** [0.550,0.825]	0.818 [0.647,0.958]	0.556 [0.312,0.783]	**0.692** [0.500,0.864]	**0.687** [0.550,0.827]

For each metric, the 95% confidence interval is shown and the highest value is highlighted in bold

**Table 3:** Response prediction performance comparison between baseline, delta and longitudinal models in the independent test set for endpoint PFS9 by evaluating AUC, ACC, SENS, SPEC, PREC and bACC, respectively

Model	Features	N test	AUC [95% CI]	ACC [95% CI]	SENS [95% CI]	SPES [95% CI]	PREC [95% CI]	bACC [95% CI]
CNN-baseline	Image data	43	0.429 [0.249,0.616]	0.674 [0.535,0.814]	1.000 [1.000,1.000]	0.000 [0.000,0.000]	0.674 [0.535,0.814]	0.500 [0.500, 0.500]
RF-baseline	Clinical data	43	0.563 [0.392,0.735]	0.581 [0.442,0.721]	0.793 [0.636,0.929]	0.143 [0.000,0.357]	0.657 [0.500,0.811]	0.468 [0.352,0.591]
RF-baseline	Radiomics	43	0.286 [0.112,0.494]	0.512 [0.372,0.651]	0.655 [0.480,0.815]	0.214 [0.000,0.455]	0.633 [0.464,0.800]	0.435 [0.303,0.576]
RF-baseline	DF-imm	43	0.541 [0.359,0.724]	0.628 [0.488,0.767]	0.759 [0.600,0.903]	0.357 [0.118,0.600]	0.710 [0.533,0.867]	0.558 [0.405,0.711]
RF-delta	Clinical data	21	0.550 [0.301,0.795]	0.524 [0.333,0.762]	0.636 [0.333,0.900]	0.400 [0.100,0.714]	0.538 [0.250,0.818]	0.518 [0.306,0.750]
RF-delta	Radiomics	36	0.598 [0.353,0.848]	0.639 [0.472,0.778]	0.680 [0.500,0.857]	**0.545** [0.231,0.857]	**0.773** [0.588,0.947]	0.613 [0.429,0.788]
RF-delta	DF-imm	36	0.525 [0.315,0.743]	0.556 [0.389,0.722]	0.760 [0.571,0.920]	0.091 [0.000,0.300]	0.655 [0.481,0.824]	0.425 [0.315,0.554]
RF-longitudinal	Clinical data	33	0.585 [0.367,0.783]	0.545 [0.364,0.697]	0.600 [0.381,0.812]	0.462 [0.182,0.727]	0.632 [0.412,0.850]	0.531 [0.360,0.698]
RF-longitudinal	Radiomics	40	0.528 [0.341,0.701]	0.558 [0.395,0.698]	0.724 [0.562,0.88]	0.214 [0.000,0.455]	0.656 [0.484,0.818]	0.469 [0.338,0.612]
RF-longitudinal	DF-imm	40	**0.702** [0.515,0.867]	**0.750** [0.625,0.875]	**0.885** [0.750,1.000]	0.500 [0.214,0.769]	0.767 [0.606,0.914]	**0.692** [0.540,0.840]

For each metric, the 95% confidence interval is shown and the highest value is highlighted in bold

### Integration of imaging and clinical data

**Fig. 5:**
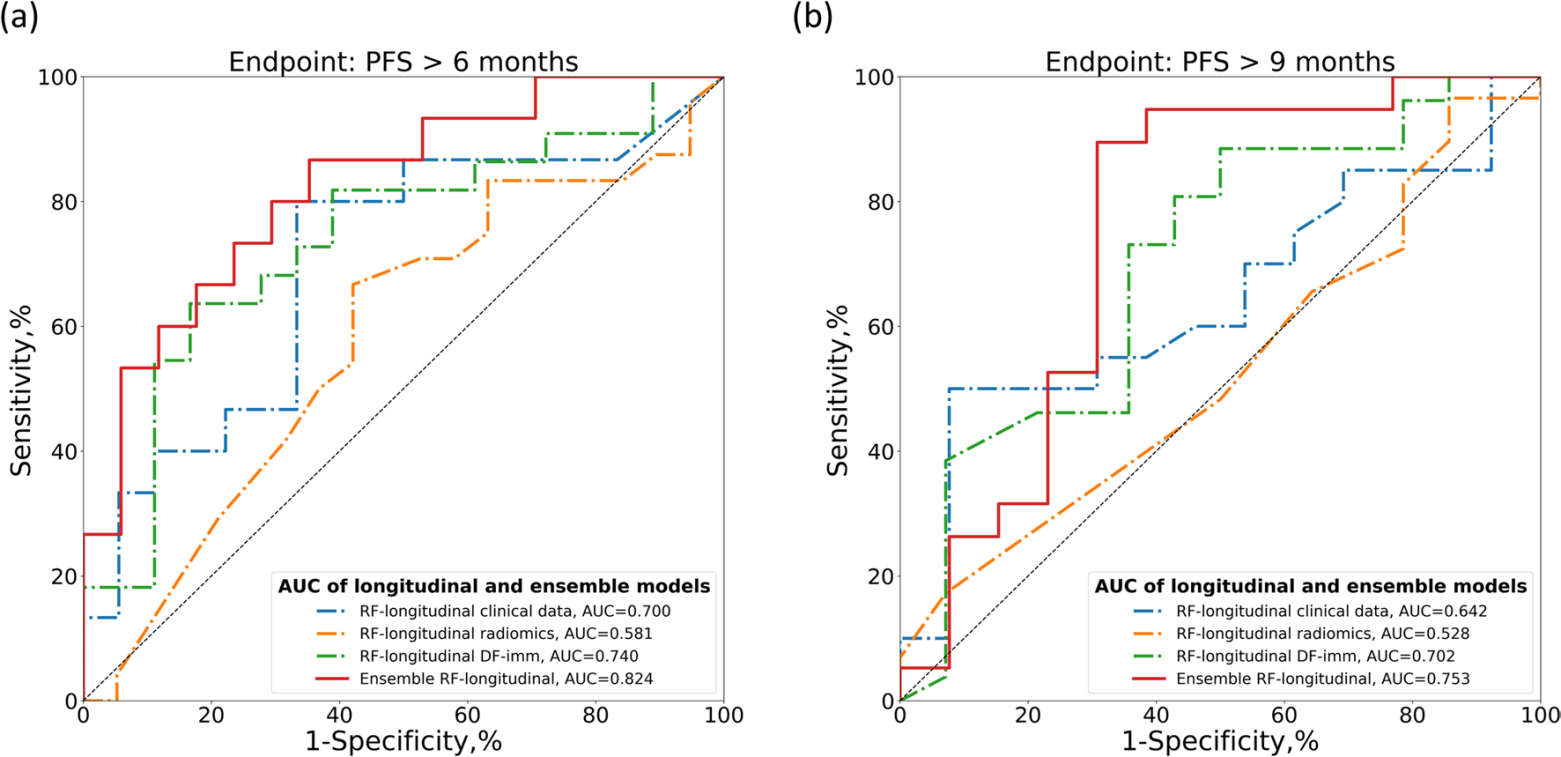
Comparisons of ROC curves of longitudinal and ensemble RF models with clinical and radiomics data. **a** ROC curves for PFS6: PFS> 6 months. **b** ROC curve for PFS9: PFS > 9 months

Table 4 shows the performance in the independent test set of the ensemble RF models that used both clinical and imaging information. The comparison with baseline and longitudinal RF models tested on the same patients is shown in Additional file 1: Tables S10 and S11 for endpoint PFS6 and PFS9, respectively. The ensemble RF-longitudinal achieved an AUC of 0.824 (95% CI: 0.658$-$0.953) for PFS6 with a 41% improvement for RF models with only clinical data (DeLong test: p-value = 0.001) and 13% for the RF model with deep features data (DeLong test: p-value = 0.013). When considering PFS9, the ensemble model achieved an AUC of 0.753 (95% CI: 0.549$-$0.931) with a 31% improvement compared to RF models with only clinical data (DeLong test: p-value = 0.053) and 5% for the RF model based on deep features data (DeLong test: p-value = 0.058) (Fig. 5). Furthermore, the ensemble models scores were significantly associated with progression-free survival and overall survival in the independent test set (6 months: C-index 4.68, 95% CI: [1.52,7.84], p-value< 0.004; 9 months: C-index 2.38, 95% CI: [0.23,4.54], p-value< 0.030). The HRs with their corresponding 95% CIs and the C-indexes of longitudinal and ensemble RF models for PFS and OS are shown in Tables 5 (endpoint PFS6) and 6 (endpoint PFS9). The integration of clinical and DF-imm data appeared to be a more robust approach compared to the radiomics or clinical models.

**Table 4:** Response prediction performance comparison between longitudinal and ensemble models in the independent test set for endpoint PFS6 and PFS9 by evaluating AUC, ACC, SENS, SPEC, PREC and bACC, respectively

Endpoint	Model	Features	N test	AUC [95% CI]	ACC [95% CI]	SENS [95% CI]	SPES [95% CI]	PREC [95% CI]	bACC [95% CI]
PFS6	Ensemble RF-baseline	DF-imm Clinical data	43	0.678 [0.513,0.836]	0.605 [0.442,0.744]	**0.875** [0.731,1.000]	0.263 [0.071,0.467]	0.600 [0.436,0.758]	0.569 [0.448,0.684]
	Ensemble RF-longitudinal	DF-imm Clinical data	32	**0.824** [0.658,0.953]	**0.750** [0.594,0.906]	0.733 [0.500,0.938]	**0.765** [0.533,0.947]	**0.733** [0.471,0.933]	**0.749** [0.594,0.897]
PFS9	Ensemble RF-baseline	DF-imm Clinical data	43	0.560 [0.377,0.731]	0.581 [0.442,0.721]	0.793 [0.643,0.933]	0.143 [0.000,0.364]	0.657 [0.487,0.811]	0.468 [0.360,0.590]
	Ensemble RF-longitudinal	DF-imm Clinical data	32	**0.753** [0.549,0.931]	**0.813** [0.656,0.938]	**0.947** [0.826,1.000]	**0.615** [0.357,0.889]	**0.783** [0.609,0.950]	**0.781** [0.631,0.923]

For each metric, the 95% confidence interval is shown and the highest value for each endpoint is highlighted in bold

**Table 5:** Hazard ratios and C-indexes of longitudinal and ensemble models trained for endpoint PFS6 to predict PFS and OS in the independent test set

		PFS			OS			
Model	Features	HR [95% CI]	p-value	C-index	HR [95% CI]		p-value	C-index
RF-longitudinal	Clinical data	1.63 [-1.00,4.25]	0.224	0.615	3.49 [0.28,6.69]		0.033	0.656
RF-longitudinal	DF-imm	3.30 [1.02,5.59]	0.005	0.687	4.31 [1.43,7.12]		0.003	0.709
Ensemble RF-longitudinal	DF-imm Clinical data	**4.68** [1.52,7.84]	**0.004**	**0.723**	**6.00** [2.27,9.73]		**0.002**	**0.768**

The highest value for each metric is highlighted in bold

**Table 6:** Hazard ratios and C-indexes of longitudinal and ensemble models trained for endpoint PFS9 to predict PFS and OS in the independent test set

Model	Features	PFS			OS			
		HR [95% CI]	p-value	C-index	HR [95% CI]		p-value	C-index
RF-longitudinal	Clinical data	0.52 [-1.16,2.20]	0.542	0.575	1.73 [-0.67,4.13]		0.157	0.613
RF-longitudinal	DF-imm	1.35 [-0.23,2.92]	0.093	0.642	1.72 [-0.18,3.62]		0.076	0.641
Ensemble RF-longitudinal	DF-imm Clinical data	**2.38** [0.23,4.54]	**0.030**	**0.685**	**2.94** [0.40,5.48]		**0.023**	**0.736**

The highest value for each metric is highlighted in bold

**Fig. 6:**
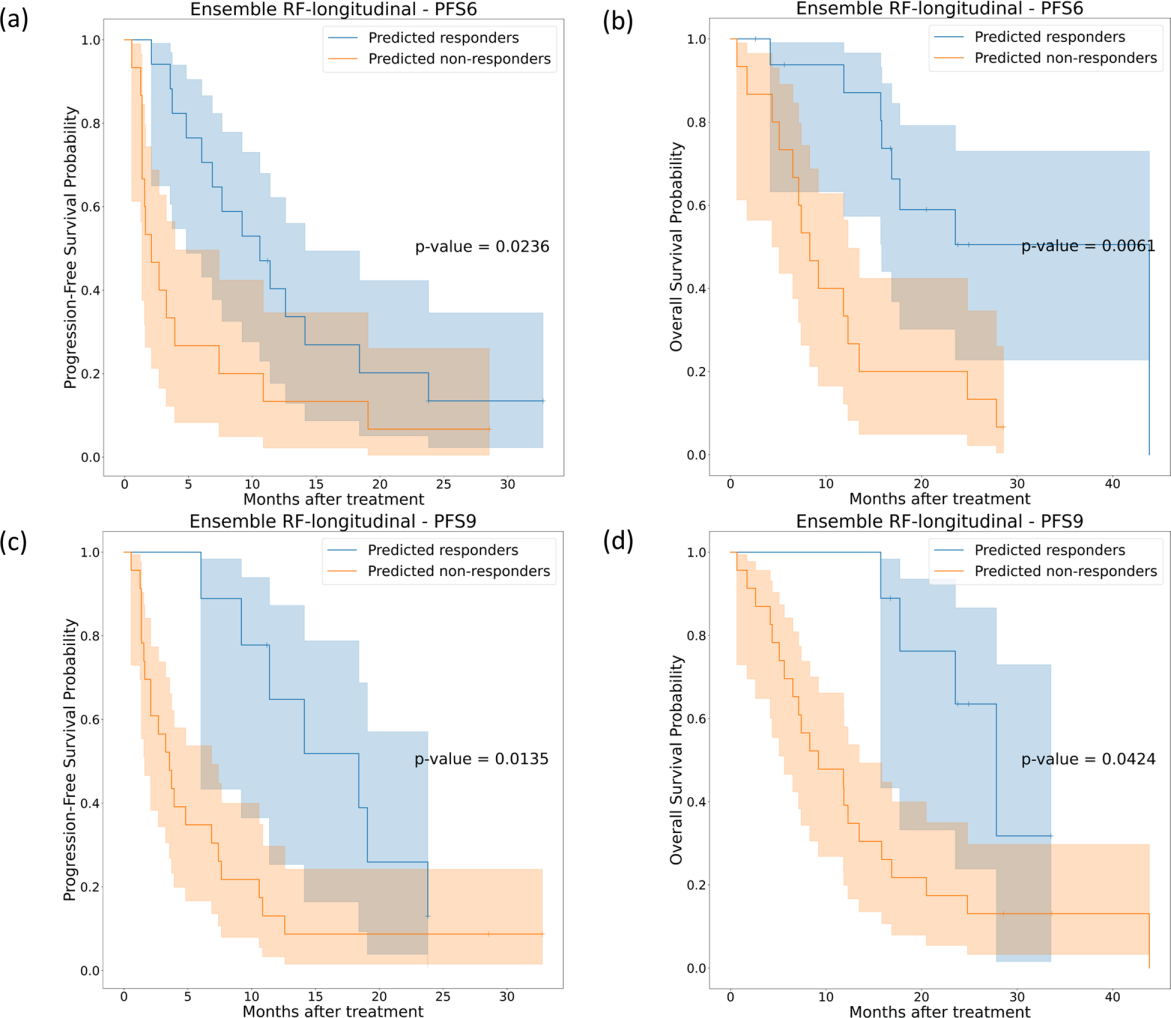
Kaplan-Meier survival curves on the independent test cohort for ensemble RF models trained for endpoint PFS6 (first row) and PFS9 (second row). **a** and **c** represent the PFS Kaplan-Meier curves, while **b** and **d** represent the OS Kaplan-Meier curves

Figure 6 shows the Kaplan-Meier survival curves for PFS and OS on the independent test set for the ensemble RF models. The ensemble RF could significantly stratify PFS and OS for both endpoints compared to the other models (p-value< 0.05). The comparisons between Kaplan-Meier curves for longitudinal RF and ensemble RF models are shown in Additional file 1: Figures S1 (endpoint PFS6) and S2 (endpoint PFS9).

### Model interpretation

**Fig. 7:**
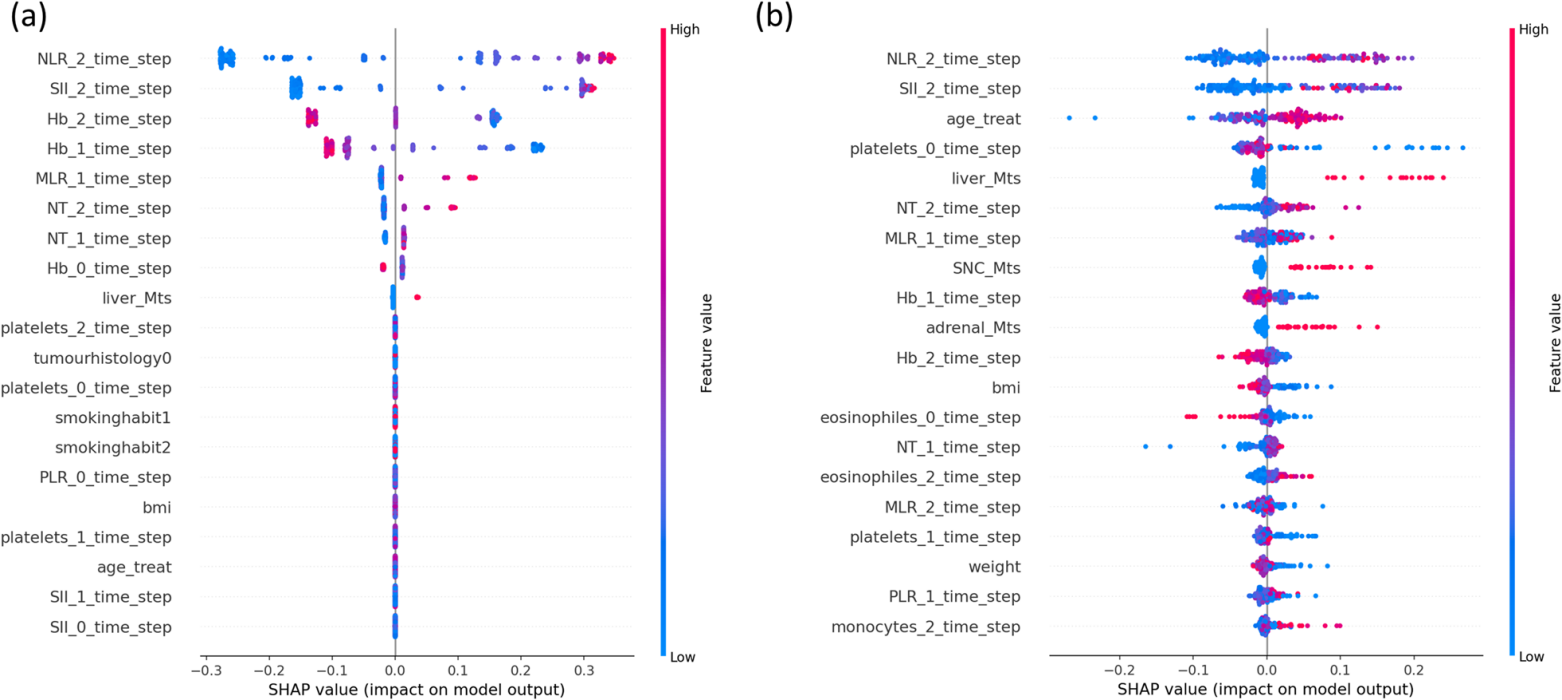
Clinical model interpretation using SHAP. The summary plots show each clinical data impact on longitudinal RF model for endpoint PFS6 (**a**) and endpoint PFS9 (**b**). A positive SHAP value indicates an increased risk of progression. Each point in the summary plot represents a patient

The SHAP algorithm was employed to visualize each feature’s contribution to producing the final prediction of the model. The SHAP algorithm was applied to the clinical model of the ensemble RF. A positive SHAP value indicated an increased risk of progression for each prediction. As observed in Fig. 7, the most important clinical variables were the neutrophils-to-lymphocytes ratio (NLR) and the systemic immune-inflammation index (SII): for both endpoints, the higher the values in the second time step (around 1–2 months after treatment), the higher the probability of progression. Moreover, the presence of liver metastases appeared to be related to a worse outcome.

## Discussion

In immuno-oncology, the traditional approach of manually measuring the size changes of the target lesions during treatment is no longer adequate because the tumor unconventionally responds to treatment [29]. Therefore, identifying unusual tumor response patterns could avoid premature treatment interruptions or ineffective prolongation. Automatic extraction of imaging biomarkers that capture changes in tumor radiophenotypes during treatment in association with clinical information can potentially aid in patient evaluation and ultimately monitor and adapt therapy dynamically.

In this two-institutional study, longitudinal information from clinical data and radiomics was used to predict clinical durable benefit at 6 and 9 months after the start of anti-PD-1/PD-L1 monoclonal antibodies treatment in advanced NSCLC patients using an ensemble approach.

A deep-learning method was used to automatically extract spatial information from CT scans without manual or semiautomatic segmentation and with the advantage of extracting features closely associated with response. Furthermore, deep-features compared to traditional radiomics may be more robust to noise variability introduced during image acquisition, making them more reproducible. Previous studies have demonstrated the ability of deep learning to capture higher-level features related to the immunotherapy response [20, 30–32]. The results of this study demonstrated that the deep features were more robust than traditional radiomics in predicting immunotherapy clinical durable benefit in advanced NSCLC, as well as in survival prediction and patient stratification. This confirms the hypothesis that deep-learning techniques allow the extraction of higher-level spatial features that are deeply related to response to treatment. They might represent properties of the tumors that are indicative of treatment response, such as changes in shape, size or intensity.

Moreover, a multiple time-point analysis was performed. Typically, only data before the start of treatment is used for prediction, without including any information during treatment. In previous studies, longitudinal data have been used to predict immunotherapy response from baseline and first follow-up CT scans [14, 15, 14, 15]. However, using data before treatment and up to four months after treatment (up to three time points per patient), we were able to improve the predictions of durable clinical benefit of immunotherapy.

To the best of our knowledge, no previous studies have demonstrated that the integration of complementary longitudinal clinical and imaging data can significantly improve immunotherapy clinical benefit prediction. The ensembles of longitudinal models with deep-radiomics (DF-imm) and clinical data significantly improved prediction performance, achieving an AUC of 0.824 for PFS6 and an AUC of 0.753 for PFS9. These models significantly stratified patients in high- and low-risk groups for both PFS and OS (p-value< 0.05), and their predictions significantly correlated with PFS (PFS6 model: C-index 0.723, p-value = 0.004; PFS9 model: C-index 0.685, p-value = 0.030) and OS (PFS6 models: C-index 0.768, p-value = 0.002; PFS9 model: C-index 0.736, p-value = 0.023). After attempting to identify any unique characteristics among the patients with better survival, we found no significant differences in their clinical data. As a result, we have determined that the accurate predictions result from the model effectively integrating information from both the deep-features and clinical variables. As a comparison, Vanguri et al. [21] showed that integrating baseline medical imaging, histopathological and genomic features (multimodal model) outperformed unimodal models, achieving an AUC of 0.80 for the immunotherapy response prediction.

The final ensemble models considered changes in imaging tumor radiophenotypes and clinical covariates during early treatment. The SHAP analysis shows that for both PFS6 and PFS9 endpoints, the most important clinical variables were the NLR and the SII. High values of NLR and SII after the second cycle of therapy were highly associated with poor prognosis probably because of a reduced antitumor effect of the immune system. This is consistent with the literature in which baseline NLR is considered a prognostic factor associated with a lower likelihood of treatment response [34], and inflammation markers, such as SII, are related to tumor growth, progression, and poor OS [35]. In our study, both NLR and SII early follow-up values are shown to be important for the clinical durable benefit of the therapy. Furthermore, the models considered that the presence of metastases in the liver before treatment was related to a worse outcome. On the other hand, higher levels of hemoglobin before and during treatment were associated with a better response to treatment.

Our study had some limitations. First, the retrospective and multi-center nature of the work implies a heterogeneity of the cohort in terms of treatment and imaging protocols. Second, the sample size of the two cohorts (FJD and CUN) was relatively large, but a relevant number of cases did not have longitudinal imaging data. Third, there was an important unbalance between responders and nonresponders for PFS9. The SMOTE technique was used to partially reduce this imbalance during the model training, but it did not result in performance comparable to the PFS6 models. To further improve the prediction of treatment response, it may be necessary to collect more data from patients with prolonged responses to treatment and/or include more time points in the analysis. Forth, the interpretation of the deep-features is often not straightforward since they are optimized to minimize the prediction error and are not designed to match human intuition or knowledge. Despite the limitations, they can still offer insights into the relationships between the tumors’ image information and response prediction and contribute to making accurate predictions. Finally, no comparison with other prognostic biomarkers was made, such as PDL1 or tumor mutational burden, due to their inaccessibility. Similarly, for the definition of radiological progression, the iRECIST criteria were not quantitatively evaluated by the radiologists, so that no comparison could have been performed. In addition, the integration of these biomarkers, as well as other new molecular parameters from liquid biopsies such as circulating tumor DNA, circulating tumor cells, circulating endothelial cells or the changes in variant allele frequencies with the deep features and clinical data used in the study, may enhance the performance of the models even further [36, 37].

## Conclusion

In conclusion, an ensemble of longitudinal deep-radiomics and clinical data has been used to predict the durable clinical benefit of immunotherapy at 6 and 9 months after treatment. Our results demonstrate that integrating multidimensional and longitudinal data improves prediction performance. The model may be used as a prognostic biomarker and decision-support tool that can assist oncologists in identifying patients for whom the therapy is effective, avoiding premature interruptions or, on the other hand, the lengthening of an ineffective treatment.

## Supplementary Information

**Additional file 1: Table S1.** Number of patients in the training and independent test set for each model considering the endpoint PFS6.** Table S2.** Number of patients in the training and independent test set for each model considering the endpoint PFS9.** Table S3.** CT image acquisition and reconstruction parameters for the two institutions involved in the study: FJD and CUN.** Table S4.** Results of the feature repeatability against segmentation and feature reproducibility. **Table S5.** Clinical variables used for the implementation of the clinical models.** Table S6.** Demographic and clinical characteristics of the patients in the baseline analysis with imaging data. P-values of no significant difference analysis (p-value > 0.05) between the training and test set after two samples T-test for continuous variables, and Chi-square test for categorical variables. SD represents the standard deviation, and Q1 and Q3 represent the first and third quartiles, respectively.** Table S7.** Demographic and clinical characteristics of the patients in the longitudinal analysis with imaging data. P-values of no significant difference analysis (p-value > 0.05) between the training and test set after two samples T-test for continuous variables, and Chi-square test for categorical variables. SD represents the standard deviation, and Q1 and Q3 represent the first and third quartiles, respectively.** Table S8.** Number of features selected for each RF model. Longitudinal models had as input the concatenation of features extracted from baseline, 1st and 2nd follow-up data (n time steps = 3). In the case of clinical models, only 12 variables had continuous values.** Table S9.** Results of the implemented models in the training set for PFS6 and PFS9. The results are presented in terms of the area under the curve ROC curve (AUC) for the 3-fold cross validation.** Table S10.** Response prediction performance comparison between longitudinal and ensemble models in the independent test set for endpoint PFS6 by evaluating AUC, ACC, SENS, SPEC, PREC and bACC, respectively. For each metric, the 95% confidence interval is shown. The highest value for each metric is highlighted in bold.** Table S11.** Response prediction performance comparison between longitudinal and ensemble models in the independent test set for endpoint PFS9 by evaluating AUC, ACC, SENS, SPEC, PREC and bACC, respectively. For each metric, the 95% confidence interval is shown and the highest value is highlighted in bold.

## Data Availability

The immunotherapy data that support the findings of this study are available from the corresponding author, BF, upon reasonable request. The data from LIDC-IDRI dataset are available in a public repository at https://wiki.cancerimagingarchive.net/pages/viewpage.action?pageId=1966254

## Abbreviations

NSCLC: Non-small cell lung cancer
CT: Computed tomography
CI: Confidence interval
RECIST: Response evaluation criteria in solid tumors
FJD: Hospital Universitario Fundación Jiménez Díaz
CUN: Clínica Universidad de Navarra
PFS: Progression-free survival
OS: Overall survival
CNN: Convolutional neural network
LIDC-IDRI: The Lung Image Database Consortium and Image Database Resource Initiation Data Set
RF: Random forest
SMOTE: Synthetic minority oversampling technique
SHAP: SHapley Additive exPlanations
ROC: Receiver operating characteristic curve
AUC: Area under the ROC curve
HR: Hazard ratio
NLR: Neutrophils-to-lymphocytes ratio
SII: Systemic immune-inflammation index

## References

[1] Gridelli C, Peters S, Mok T, Forde PM, Reck M, Attili I, de Marinis F (2022). First-line immunotherapy in advanced non-small-cell lung cancer patients with ECOG performance status 2: results of an international expert panel meeting by the italian association of thoracic oncology. ESMO Open..

[2] Doroshow DB, Sanmamed MF, Hastings K, Politi K, Rimm DL, Chen L, Melero I, Schalper KA, Herbst RS (2019). Immunotherapy in non-small cell lung cancer: facts and hopes. Clin Cancer Res.

[3] Patel SA, Weiss J (2020). Advances in the treatment of non-small cell lung cancer: immunotherapy. Clin Chest Med.

[4] Broderick SR (2020). Adjuvant and neoadjuvant immunotherapy in non-small cell lung cancer. Thorac Surg Clin.

[5] Paz-Ares L, Ciuleanu T-E, Cobo M, Schenker M, Zurawski B, Menezes J, Richardet E, Bennouna J, Felip E, Juan-Vidal O, Alexandru A, Sakai H, Lingua A, Salman P, Souquet P-J, De Marchi P, Martin C, Pérol M, Scherpereel A, Lu S, John T, Carbone DP, Meadows-Shropshire S, Agrawal S, Oukessou A, Yan J, Reck M (2021). First-line nivolumab plus ipilimumab combined with two cycles of chemotherapy in patients with non-small-cell lung cancer (CheckMate 9LA): an international, randomised, open-label, phase 3 trial. Lancet Oncol.

[6] Kanwal B, Biswas S, Seminara RS, Jeet C (2018). Immunotherapy in advanced non-small cell lung cancer patients: ushering chemotherapy through the checkpoint inhibitors?. Cureus.

[7] Blons H, Garinet S, Laurent-Puig P, Oudart J-B (2019). Molecular markers and prediction of response to immunotherapy in non-small cell lung cancer, an update. J Thorac Dis.

[8] Suresh K, Naidoo J, Lin CT, Danoff S (2018). Immune checkpoint immunotherapy for non-small cell lung cancer: benefits and pulmonary toxicities. Chest.

[9] Dong A, Zhao Y, Li Z, Hu H (2021). PD-L1 versus tumor mutation burden: Which is the better immunotherapy biomarker in advanced non-small cell lung cancer?. J Gene Med.

[10] Bai R, Lv Z, Xu D, Cui J (2020). Predictive biomarkers for cancer immunotherapy with immune checkpoint inhibitors. Biomark Res.

[11] Eisenhauer EA, Therasse P, Bogaerts J, Schwartz LH, Sargent D, Ford R, Dancey J, Arbuck S, Gwyther S, Mooney M, Rubinstein L, Shankar L, Dodd L, Kaplan R, Lacombe D, Verweij J (2009). New response evaluation criteria in solid tumours: revised RECIST guideline (version 1.1). Eur J Cancer.

[12] Seymour L, Bogaerts J, Perrone A, Ford R, Schwartz LH, Mandrekar S, Lin NU, Litière S, Dancey J, Chen A, Hodi FS, Therasse P, Hoekstra OS, Shankar LK, Wolchok JD, Ballinger M, Caramella C, de Vries EGE (2017). iRECIST: guidelines for response criteria for use in trials testing immunotherapeutics. Lancet Oncol.

[13] Gillies RJ, Kinahan PE, Hricak H (2016). Radiomics: images are more than pictures, they are data. Radiology.

[14] Gong J, Bao X, Wang T, Liu J, Peng W, Shi J, Wu F, Gu Y (2022). A short-term follow-up CT based radiomics approach to predict response to immunotherapy in advanced non-small-cell lung cancer. Oncoimmunology.

[15] Khorrami M, Prasanna P, Gupta A, Patil P, Velu PD, Thawani R, Corredor G, Alilou M, Bera K, Fu P, Feldman M, Velcheti V, Madabhushi A (2020). Changes in CT radiomic features associated with lymphocyte distribution predict overall survival and response to immunotherapy in non-small cell lung cancer. Cancer Immunol Res.

[16] Tunali I, Gray JE, Qi J, Abdalah M, Jeong DK, Guvenis A, Gillies RJ, Schabath MB (2019). Novel clinical and radiomic predictors of rapid disease progression phenotypes among lung cancer patients treated with immunotherapy: An early report. Lung Cancer (Amsterdam, Netherlands).

[17] Trebeschi S, Bodalal Z, Boellaard TN, Tareco Bucho TM, Drago SG, Kurilova I, Calin-Vainak AM, DelliPizzi A, Muller M, Hummelink K, Hartemink KJ, Nguyen-Kim TDL, Smit EF, Aerts HJWL, Beets-Tan RGH (2021). Prognostic value of deep learning-mediated treatment monitoring in lung cancer patients receiving immunotherapy. Front Oncol.

[18] Trebeschi S, Drago SG, Birkbak NJ, Kurilova I, Cǎlin AM, DelliPizzi A, Lalezari F, Lambregts DMJ, Rohaan MW, Parmar C, Rozeman EA, Hartemink KJ, Swanton C, Haanen JBAG, Blank CU, Smit EF, Beets-Tan RGH, Aerts HJWL (2019). Predicting response to cancer immunotherapy using noninvasive radiomic biomarkers. Ann Oncol Off J Eur Soc Med Oncol.

[19] Mu W, Jiang L, Shi Y, Tunali I, Gray JE, Katsoulakis E, Tian J, Gillies RJ, Schabath MB (2021). Non-invasive measurement of PD-L1 status and prediction of immunotherapy response using deep learning of PET/CT images. J Immunother Cancer.

[20] Tian P, He B, Mu W, Liu K, Liu L, Zeng H, Liu Y, Jiang L, Zhou P, Huang Z, Dong D, Li W (2021). Assessing PD-L1 expression in non-small cell lung cancer and predicting responses to immune checkpoint inhibitors using deep learning on computed tomography images. Theranostics.

[21] Vanguri RS, Luo J, Aukerman AT, Egger JV, Fong CJ, Horvat N, Pagano A, Araujo-Filho JDAB, Geneslaw L, Rizvi H, Sosa R, Boehm KM, Yang S-R, Bodd FM, Ventura K, Hollmann TJ, Ginsberg MS, Gao J, MSK MIND Consortium, Vanguri R, Hellmann MD, Sauter JL, Shah SP. Multimodalz integration of radiology, pathology and genomics for prediction of response to PD-(L)1 blockade in patients with non-small cell lung cancer. Nat Cancer 2022; 3(10): 1151-1164 10.1038/s43018-022-00416-8 PMC9586871 36038778

[22] Dercle L, McGale J, Sun S, Marabelle A, Yeh R, Deutsch E, Mokrane F-Z, Farwell M, Ammari S, Schoder H, Zhao B, Schwartz LH (2022). Artificial intelligence and radiomics: fundamentals, applications, and challenges in immunotherapy. J Immunother Cancer..

[23] Fedorov A, Beichel R, Kalpathy-Cramer J, Finet J, Fillion-Robin J-C, Pujol S, Bauer C, Jennings D, Fennessy F, Sonka M, Buatti J, Aylward S, Miller JV, Pieper S, Kikinis R (2012). 3D slicer as an image computing platform for the quantitative imaging network. Magn Reson Imaging.

[24] van Griethuysen JJM, Fedorov A, Parmar C, Hosny A, Aucoin N, Narayan V, Beets-Tan RGH, Fillion-Robin J-C, Pieper S, Aerts HJWL (2017). Computational radiomics system to decode the radiographic phenotype. Cancer Res.

[25] Larue RTHM, van Timmeren JE, de Jong EEC, Feliciani G, Leijenaar RTH, Schreurs WMJ, Sosef MN, Raat FHPJ, van der Zande FHR, Das M, van Elmpt W, Lambin P (2017). Influence of gray level discretization on radiomic feature stability for different ct scanners, tube currents and slice thicknesses: a comprehensive phantom study. Acta Oncol.

[26] Causey JL, Zhang J, Ma S, Jiang B, Qualls JA, Politte DG, Prior F, Zhang S, Huang X (2018). Highly accurate model for prediction of lung nodule malignancy with CT scans. Sci Rep.

[27] Armato SG, McLennan G, Bidaut L, McNitt-Gray MF, Meyer CR, Reeves AP, Zhao B, Aberle DR, Henschke CI, Hoffman EA, Kazerooni EA, MacMahon H, Van Beeke EJR, Yankelevitz D, Biancardi AM, Bland PH, Brown MS, Engelmann RM, Laderach GE, Max D, Pais RC, Qing DPY, Roberts RY, Smith AR, Starkey A, Batrah P, Caligiuri P, Farooqi A, Gladish GW, Jude CM, Munden RF, Petkovska I, Quint LE, Schwartz LH, Sundaram B, Dodd LE, Fenimore C, Gur D, Petrick N, Freymann J, Kirby J, Hughes B, Casteele AV, Gupte S, Sallamm M, Heath MD, Kuhn MH, Dharaiya E, Burns R, Fryd DS, Salganicoff M, Anand V, Shreter U, Vastagh S, Croft BY (2011). The lung image database consortium (LIDC) and image database resource initiative (IDRI): a completed reference database of lung nodules on CT scans. Med Phys.

[28] Lundberg SM, Lee SI, Guyon I, Luxburg UV, Bengio S, Wallach H, Fergus R, Vishwanathan S, Garnett R (2017). A unified approach to interpreting model predictions. Advances in neural information processing systems.

[29] Borcoman E, Kanjanapan Y, Champiat S, Kato S, Servois V, Kurzrock R, Goel S, Bedard P, Le Tourneau C (2019). Novel patterns of response under immunotherapy. Ann Oncol.

[30] Mu W, Jiang L, Shi Y, Tunali I, Gray JE, Katsoulakis E, Tian J, Gillies RJ, Schabath MB. Non-invasive measurement of PD-L1 status and prediction of immunotherapy response using deep learning of pet/ct images. J Immunother Cancer. 2021;9(6):10.1136/jitc-2020-002118 PMC8211060 34135101

[31] He B, Dong D, She Y, Zhou C, Fang M, Zhu Y, Zhang H, Huang Z, Jiang T, Tian J, Chen C (2020). Predicting response to immunotherapy in advanced non-small-cell lung cancer using tumor mutational burden radiomic biomarker. J Immunother Cancer.

[32] Trebeschi S, Bodalal Z, Boellaard TN, Tareco Bucho TM, Drago SG, Kurilova I, Calin-Vainak AM, Delli Pizzi A, Muller M, Hummelink K, Hartemink KJ, Nguyen-Kim TDL, Smit EF, Aerts HJWL, Beets-Tan RGH (2021). Prognostic value of deep learning-mediated treatment monitoring in lung cancer patients receiving immunotherapy. Front Oncol.

[33] Liu Y, Wu M, Zhang Y, Luo Y, He S, Wang Y, Chen F, Liu Y, Yang Q, Li Y, Wei H, Zhang H, Jin C, Lu N, Li W, Wang S, Guo Y, Ye Z (2021). Imaging biomarkers to predict and evaluate the effectiveness of immunotherapy in advanced non-small-cell lung cancer. Front Oncol.

[34] Valero C, Lee M, Hoen D, Weiss K, Kelly DW, Adusumilli PS, Paik PK, Plitas G, Ladanyi M, Postow MA, Ariyan CE, Shoushtari AN, Balachandran VP, Hakimi AA, Crago AM, LongRoche KC, Smith JJ, Ganly I, Wong RJ, Patel SG, Shah JP, Lee NY, Riaz N, Wang J, Zehir A, Berger MF, Chan TA, Seshan VE, Morris LGT (2021). Pretreatment neutrophil-to-lymphocyte ratio and mutational burden as biomarkers of tumor response to immune checkpoint inhibitors. Nat Commun.

[35] Fu F, Deng C, Wen Z, Gao Z, Zhao Y, Han H, Zheng S, Wang S, Li Y, Hu H, Zhang Y, Chen H (2021). Systemic immune-inflammation index is a stage-dependent prognostic factor in patients with operable non-small cell lung cancer. Transl Lung Cancer Res.

[36] Sinoquet L, Jacot W, Quantin X, Alix-Panabières C (2022). Liquid biopsy and immuno-oncology for advanced nonsmall cell lung cancer. Clin Chem.

[37] Kato S, Li B, Adashek JJ, Cha SW, Bianchi-Frias D, Qian D, Kim L, So TW, Mitchell M, Kamei N, Hoiness R, Hoo J, Gray PN, Iyama T, Kashiwagi M, Lu H-M, Kurzrock R (2022). Serial changes in liquid biopsy-derived variant allele frequency predict immune checkpoint inhibitor responsiveness in the pan-cancer setting. OncoImmunology.

