# Supplementary material for: Integration of longitudinal deep-radiomics and clinical data improves the prediction of durable benefits to anti-PD-1/PD-L1 immunotherapy in advanced NSCLC patients
Source: J Transl Med. 2023 Mar 5;21:174. doi: 10.1186/s12967-023-04004-x (PMC9985838; doi:10.1186/s12967-023-04004-x)
Supplement: Supplementary file 1 — Additional file 1: Table S1. Number of patients in the training and independent test set for each model considering the endpoint PFS6. Table S2. Number of patients in the training and independent test set for each model considering the endpoint PFS9. Table S3. CT image acquisition and reconstruction parameters for the two institutions involved in the study: FJD and CUN. Table S4. Results of the feature repeatability against segmentation and feature reproducibility. Table S5. Clinical variables used for the implementation of the clinical models. Table S6. Demographic and clinical characteristics of the patients in the baseline analysis with imaging data. P-values of no significant difference analysis (p-value > 0.05) between the training and test set after two samples T-test for continuous variables, and Chi-square test for categorical variables. SD represents the standard deviation, and Q1 and Q3 represent the first and third quartiles, respectively. Table S7. Demographic and clinical characteristics of the patients in the longitudinal analysis with imaging data. P-values of no significant difference analysis (p-value > 0.05) between the training and test set after two samples T-test for continuous variables, and Chi-square test for categorical variables. SD represents the standard deviation, and Q1 and Q3 represent the first and third quartiles, respectively. Table S8. Number of features selected for each RF model. Longitudinal models had as input the concatenation of features extracted from baseline, 1st and 2nd follow-up data (n time steps = 3). In the case of clinical models, only 12 variables had continuous values. Table S9. Results of the implemented models in the training set for PFS6 and PFS9. The results are presented in terms of the area under the curve ROC curve (AUC) for the 3-fold cross validation. Table S10. Response prediction performance comparison between longitudinal and ensemble models in the independent test set for endpoint PFS6 by evaluating AU [file 12967_2023_4004_MOESM1_ESM.pdf]

Supplementary Material

S1. Train-test split

PFS6	Train set			Test set		
**Data**	**RF-baseline**	**RF-delta**	**RF-longitudinal**	**RF-baseline**	**RF-delta**	**RF-longitudinal**
Only clinical data	221	114	167	43	21	33
Only imaging data	128	96	109	43	36	40
Clinical and imaging data	128	-	92	43	-	32

Supplementary Table S1 Number of patients in the training and independent test set for each model considering the endpoint PFS6.

PFS9	Train set			Test set		
**Data**	**RF-baseline**	**RF-delta**	**RF-longitudinal**	**RF-baseline**	**RF-delta**	**RF-longitudinal**
Only clinical data	216	112	163	43	21	33
Only imaging data	125	93	106	43	36	40
Clinical and imaging data	125	-	90	43	-	32

Supplementary Table S2 Number of patients in the training and independent test set for each model considering the endpoint PFS9.

S2. Imaging metadata

All CT images were acquired after contrast injection during a patient inspiratory breath hold, following the contrast-enhanced CT chest protocol.

Metadata	Value range	FJD	CUN
Manufacturer	[Siemens, Toshiba, GE Medical Systems, Philips]	[Siemens, Toshiba, Philips]	[Siemens, Toshiba, GE Medical Systems]
kVp	[80, 140]	[80, 140]	[80, 140]
Current tube	[48, 1481]	[60, 1481]	[48, 998]
Pixel Spacing	[0.359, 1.523]	[0.359, 0.975]	[0.525, 1.523]
Slice thickness	[0.625, 5.0]	[0.900, 3.0]	[0.625, 5.0]

Supplementary Table S3 CT image acquisition and reconstruction parameters for the two institutions involved in the study: FJD and CUN.

S3. Radiomics Reproducibility Analysis

Many studies have shown evidence that radiomics features are influenced by image

acquisition parameters and image segmentation. For this reason, we performed a reproducibility analysis to select only stable features with respect to image acquisition parameters and changes in segmentations.
Feature repeatability against segmentation was verified using: the QIN Lung CT Segmentation dataset [1] and a random subset of the immunotherapy dataset. In the first case, two automatic segmentations with two different algorithms for each nodule were considered. In the second case, an experienced radiologist refined two segmentations of the same nodule obtained with two different modules of syngo.via software. A total of 56 nodules were analyzed.
Feature reproducibility was assessed through a test-retest analysis using the Reference Image Database to Evaluate Therapy Response (RIDER) dataset [2]. The dataset included 31 patients who underwent two chest CT scans, acquired 15 minutes apart with the same image protocol.
Lin's concordance correlation coefficient (CCC) was used to assess feature repeatability and reproducibility. Features presenting a high CCC (≥ 0.85) for both tests were considered stable and used for further analysis.

	**N features**	**N stable features**
Repeatability	1365	189 (15%)
Reproducibility	1365	914 (68%)
Total Stable	1365	173 (13%)

Supplementary Table S4 Results of the feature repeatability against segmentation and feature reproducibility.

S4. Characterization Dataset

In addition to the immunotherapy dataset, a dataset was considered to train a deep learning model on nodule characteristics for feature extraction. Benign and confirmed malignant nodules were collected from 719 patients of The Lung Image Database Consortium and Image Database Resource Initiation Data Set (LIDC-IDRI), which consists of annotated chest CT scans for lung cancer screening. Fourteen patients who did not meet the inclusion criteria of the immunotherapy dataset were also added to this collection for a total of 733 patients. Their nodules were all malignant. Given that each patient may have more than one nodule, the characterization dataset contained 1,528 nodules, 1024 of which were benign and 504 malignant.

S5. Clinical data

Clinical data were extracted from patient electronic medical records and blood tests before and throughout treatment. The electronic medical record was searched to retrieve information about demographics, tumor histology, smoking habits, stage of disease, presence of metastases per site prior to the treatment, excess postexercise oxygen consumption (EPOC), etc. Hematology data were obtained from the blood test performed after the second and third cycles of immunotherapy. They included: platelets, lymphocytes, monocytes, eosinophils, hemoglobin, Neutrophil absolute count (NT), Neutrophil-to-lymphocyte ratio (NLR), Monocyte-to-lymphocyte ratio (MLR), Platelet-to-lymphocyte ratio (PLR), Systemic immune-inflammation index (SII).

Clinical variables

**Categorical variables**	**Continuous variables**	**Formula**
Sex	Platelets (cells/microL)	
Age	Lymphocytes (cells/microL)	
Weight	Monocytes (cells/microL)	
Height	Eosinophils (cells/microL)	
BMI	Hemoglobin (Hb, g/dL)	
Surgery	Neutrophil absolute count (NT, cells/microL)	
Smoking	Neutrophil-to-lymphocyte ratio (NLR)	
Tumour histology	Monocyte-to-lymphocyte ratio (MLR)	
Steroids	Platelet-to-lymphocyte ratio (PLR)	
Antibiotics	Systemic immune-inflammation index (SII)	platelets x neutrophils/lymphocytes (cells/µL)
EPOC		
SNC_Metastases		
Adrenal_ Metastases		
Liver_ Metastases		
Bone_ Metastases		

Supplementary Table S5 Clinical variables used for the implementation of the clinical models.

S6. Demographic and clinical characteristics: patients with image data

Characteristic	All Patients (N = 171)	Train set (N = 128)	Test set (N = 43)	p-value
**PFS, mean (SD)**	8.8 (10.3)	9.2 (10.9)	7.6 (8.1)	0.289
**OS, mean (SD)**	13.0 (11.3)	12.8 (11.6)	13.5 (10.5)	0.728
**Status**				
Alive	68 (39.8%)	52 (40.6%)	16 (37.2)	0.829
Dead	103 (60.2%)	76 (59.4%)	27 (62.8)	
**Response**				
Non-responders	94 (55.0%)	70 (54.7%)	24 (55.8 %)	1.000
Responders	77 (45.0%)	58 (45.3%)	19 (44.2%)	
**Progression**				
No progression	28 (16.4%)	23 (18.0%)	5 (11.6%)	0.463
Progression	143 (83.6%)	105 (82.0%)	38 (88.4%)	
**Age, median [Q1,Q3]**	67.0 [60.0,72.0]	66.0 [57.8,71.2]	67.0 [60.5,72.5]	0.529
**Sex**				
Female	56 (32.7%)	42 (32.8%)	14 (32.6%)	1.000
Male	115 (67.3%)	86 (67.2%)	29 (67.4%)	
**IPA, mean (SD)**	45.1 (33.0)	45.0 (33.6)	45.4 (31.5)	0.948
**Smoking**				
Current smoker	31 (18.2%)	26 (20.3%)	5 (11.9%)	0.465
Former smoker	125 (73.5%)	92 (71.9%)	33 (78.6%)	
Non-smoker	14(8.2%)	10 (7.8%)	4 (9.5%)	

**Tumour Histology**				
Adenocarcinoma	129 (75.4%)	96 (75.0%)	33 (76.7%)	0.886
Epidermoid carcinoma	36 (21.1%)	27 (21.1%)	9 (20.9%)	
Other	6 (3.5%)	5 (3.9%)	1 (2.3%)	
**PDL1, mean (SD)**	0.4 (0.4)	0.4 (0.4)	0.4 (0.4)	0.817
**Surgery**				
No	146 (85.4%)	109 (85.2%)	37 (86.0%)	1.000
Yes	25 (14.6%)	19 (14.8%)	6 (14.0%)	
**Treatment**				
Combined Immunological Agents	26 (15.2%)	16 (12.5%)	10 (23.3%)	0.289
Immunotherapy+Chemotherapy	30 (17.5%)	21 (16.4%)	9 (20.9%)	
Immunotherapy+Radiotherapy	15 (8.8%)	13 (10.2%)	2 (4.7%)	
Monotherapy	98 (57.3%)	76 (59.4%)	22 (51.2%)	
Other	2 (1.2%)	2 (1.6%)		

Supplementary Table S6 Demographic and clinical characteristics of the patients in the baseline analysis with imaging data. P-values of no significant difference analysis (p-value > 0.05) between the training and test set after two samples T-test for continuous variables, and Chi-square test for categorical variables. SD represents the standard deviation, and Q1 and Q3 represent the first and third quartiles, respectively.

Characteristic	All Patients (N = 149)	Train set (N = 109)	Test set (N = 40)	P-Value
**PFS, mean (SD)**	9.7 (10.7)	10.4 (11.4)	7.9 (8.3)	0.147
**OS, mean (SD)**	14.4 (11.4)	14.5 (11.8)	14.1 (10.5)	0.842
**Status**				
Alive	64 (43.0%)	49 (45.0%)	15 (37.5%)	0.530
Dead	85 (57.0%)	60 (55.0%)	25 (62.5%)	
**Response**				
Non-responders	74 (49.7%)	52 (47.7%)	22 (55.0%)	0.546
Responders	75 (50.3%)	57 (52.3%)	18 (45.0%)	
**Progression**				
No progression	28 (18.8%)	23 (21.1%)	5 (12.5%)	0.340
Progression	121 (81.2%)	86 (78.9%)	35 (87.5%)	
**Age, median [Q1,Q3]**	65.0 [59.0,71.0]	65.0 [57.0,71.0]	65.5 [60.0,71.2]	0.622
**Sex**				
Female	48 (32.2%)	34 (31.2%)	14 (35.0%)	0.808
Male	101 (67.8%)	75 (68.8%)	26 (65.0%)	
**IPA, mean (SD)**	45.5 (32.4)	45.4 (32.7)	45.9 (31.7)	0.928
**Smoking**				
Current smoker	26 (17.6%)	21 (19.3%)	5 (12.8%)	0.662
Former smoker	111 (75.0%)	80 (73.4%)	31 (79.5%)	
Non-smoker	11 (7.4%)	8 (7.3%)	3 (7.7%)	
**Tumour Histology**				

Adenocarcinoma	112 (75.2%)	81 (74.3%)	31 (77.5%)	0.830
Epidermoid carcinoma	31 (20.8%)	23 (21.1%)	8 (20.0%)	
Other	6 (4.0%)	5 (4.6%)	1 (2.5%)	
**PDL1, mean (SD)**	0.4 (0.4)	0.4 (0.4)	0.4 (0.4)	0.598
**Surgery**				
No	127 (85.2%)	93 (85.3%)	34 (85.0%)	1.000
Yes	22 (14.8%)	16 (14.7%)	6 (15.0%)	
**Treatment**				
Combined Immunological Agents	25 (16.8%)	16 (14.7%)	9 (22.5%)	0.551
Immunotherapy+Chemotherapy	30 (20.1%)	21 (19.3%)	9 (22.5%)	
Immunotherapy+Radiotherapy	15 (10.1%)	13 (11.9%)	2 (5.0%)	
Monotherapy	78 (52.3%)	58 (53.2%)	20 (50.0%)	
Other	1 (0.7%)	1 (0.9%)	0(0%)	

Supplementary Table S7 Demographic and clinical characteristics of the patients in the longitudinal analysis with imaging data. P-values of no significant difference analysis (p-value > 0.05) between the training and test set after two samples T-test for continuous variables, and Chi-square test for categorical variables. SD represents the standard deviation, and Q1 and Q3 represent the first and third quartiles, respectively.

S7. Number of features selected for each model

			PFS6	PFS9
**Model**	**Feature type**	**N features**	**N selected**	**N selected**
RF-baseline	Radiomics	173	32	91
RF-delta	Radiomics	173	110	109
RF-longitudinal	Radiomics	173 * 3 time steps	235	174
RF-baseline	DF-imm	500	31	32
RF-delta	DF-imm	500	368	214
RF-longitudinal	DF-imm	500 * 3 time steps	50	137
RF-baseline	Clinical data	27	21	27
RF-delta	Clinical data	27	26	27
RF-longitudinal	Clinical data	15 + 12 * 3 time steps	9	48

Supplementary Table S8 Number of features selected for each RF model. Longitudinal models had as input the concatenation of features extracted from baseline, 1st and 2nd follow-up data (n time steps = 3). In the case of clinical models, only 12 variables had continuous values.

S8. Results of the implemented models in the training set

			PFS6		PFS9	
Model	Split	Features	N train	CV Mean AUC	N train	CV Mean AUC
RF-baseline	Train	Radiomics	128	0.635 ± 0.014	125	0.791 ± 0.058
RF-delta	Train	Radiomics	96	0.766±0.040	93	0.790 ± 0.001
RF-longitudinal	Train	Radiomics	109	0.656 ± 0.116	106	0.807 ± 0.046
RF-baseline	Train	DF-imm	128	0.668 ± 0.052	125	0.837 ± 0.051
RF-delta	Train	DF-imm	96	0.689±0.128	93	0.829 ± 0.030
RF-longitudinal	Train	DF-imm	109	0.710 ± 0.113	106	0.802 ± 0.077
RF-baseline	Train	Clinical data	221	0.700 ± 0.050	216	0.835 ± 0.032
RF-delta	Train	Clinical data	114	0.593 ± 0.071	112	0.744 ± 0.067
RF-longitudinal	Train	Clinical data	167	0.731 ± 0.030	163	0.796 ± 0.044

Supplementary Table S9 Results of the implemented models in the training set for PFS6 and PFS9. The results are presented in terms of the area under the curve ROC curve (AUC) for the 3-fold cross validation.

S9. Comparisons RF models with radiomics and clinical data

Model	Features	N test	AUC	ACC	SENS	SPEC	PREC	bACC
RF-baseline	Clinical data	43	0.667 [0.485,0.833]	0.651 [0.512,0.791]	0.833 [0.667,0.962]	0.421 [0.2,0.65]	0.645 [0.48,0.812]	0.627 [0.488,0.774]
RF-baseline	DF-imm	43	0.588 [0.409,0.767]	0.558 [0.419,0.698]	0.833 [0.679,0.96]	0.211 [0.05,0.417]	0.571 [0.406,0.735]	0.522 [0.403,0.638]
RF-longitudinal	Clinical data	32	0.586 [0.413,0.753]	0.594 [0.406,0.750]	0.467 [0.200,0.733]	0.706 [0.467,0.909]	0.583 [0.300,0.867]	0.586 [0.417,0.75]
RF-longitudinal	DF-imm	32	0.727 [0.576,0.875]	0.719 [0.562,0.875]	0.867 [0.667,1.0]	0.588 [0.333,0.833]	0.727 [0.575,0.875]	0.650 [0.429,0.857]
Ensemble RF-baseline	DF-imm Clinical data	43	0.678 [0.513,0.836]	0.605 [0.442,0.744]	**0.875** [0.731,1.0]	0.263 [0.071,0.467]	0.600 [0.436,0.758]	0.569 [0.448,0.684]
Ensemble RF-longitudinal	DF-imm Clinical data	32	**0.824** [0.658,0.953]	**0.750** [0.594,0.906]	0.733 [0.500,0.938]	**0.765** [0.533,0.947]	**0.733** [0.471,0.933]	**0.749** [0.594,0.897]

Supplementary Table S10 Response prediction performance comparison between longitudinal and ensemble models in the independent test set for endpoint PFS6 by evaluating AUC, ACC, SENS, SPEC, PREC and bACC, respectively. For each metric, the 95% confidence interval is shown. The highest value for each metric is highlighted in bold.

Model	Features	N test	AUC	ACC	SENS	SPEC	PREC	bACC
RF-baseline	Clinical data	43	0.563 [0.392,0.735]	0.581 [0.442,0.721]	0.793 [0.636,0.929]	0.143 [0.0,0.357]	0.657 [0.5,0.811]	0.468 [0.352,0.591]
RF-baseline	DF-imm	43	0.541 [0.359,0.724]	0.628 [0.488,0.767]	0.759 [0.6,0.903]	0.357 [0.118,0.6]	0.710 [0.533,0.867]	0.558 [0.405,0.711]
RF-longitudinal	Clinical data	32	0.573 [0.4,0.742]	0.531 [0.375,0.688]	0.579 [0.353,0.789]	0.462 [0.188,0.727]	0.611 [0.4,0.842]	0.52 [0.333,0.697]
RF-longitudinal	DF-imm	32	0.717 [0.558,0.865]	0.750 [0.594,0.875]	0.895 [0.737,1.0]	0.538 [0.273,0.833]	0.739 [0.55,0.913]	0.717 [0.562,0.869]
Ensemble RF-baseline	DF-imm Clinical data	43	0.560 [0.377,0.731]	0.581 [0.442,0.721]	0.793 [0.643,0.933]	0.143 [0.0,0.364]	0.657 [0.487,0.811]	0.468 [0.36,0.59]
Ensemble RF-longitudinal	DF-imm Clinical data	32	**0.753** [0.549,0.931]	**0.813** [0.656,0.938]	**0.947** [0.826,1.0]	**0.615** [0.357,0.889]	**0.783** [0.609,0.95]	**0.781** [0.631,0.923]

Supplementary Table S11 Response prediction performance comparison between longitudinal and ensemble models in the independent test set for endpoint PFS9 by evaluating AUC, ACC, SENS, SPEC, PREC and bACC, respectively. For each metric, the 95% confidence interval is shown and the highest value is highlighted in bold.

S10. Comparisons Kaplan-Meier survival curves

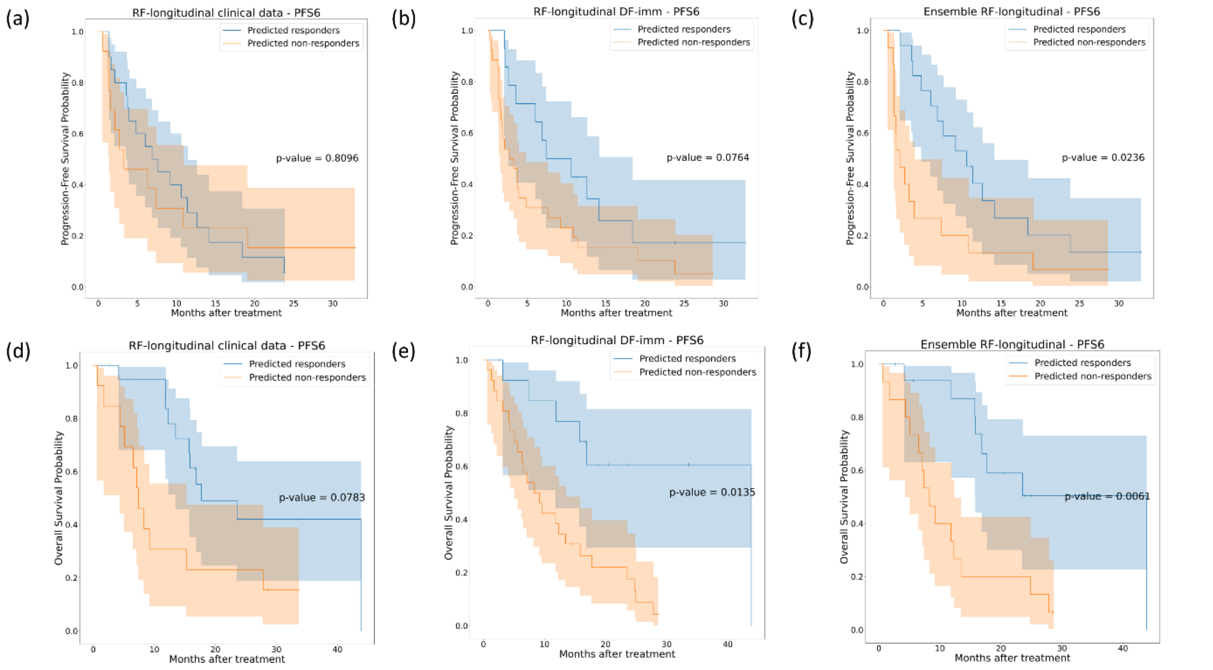

Supplementary Figure S1 Kaplan-Meier survival curves on the independent test cohort for longitudinal RF based on clinical data ((a) and (d)), longitudinal RF with deep features ((b) and (e)) and ensemble RF ((c) and (f)) trained for endpoint PFS6, according to risk groups based on each models' predictions. The first row represents the progression-free survival Kaplan-Meier curves, while the second row represents the overall survival Kaplan-Meier curves.

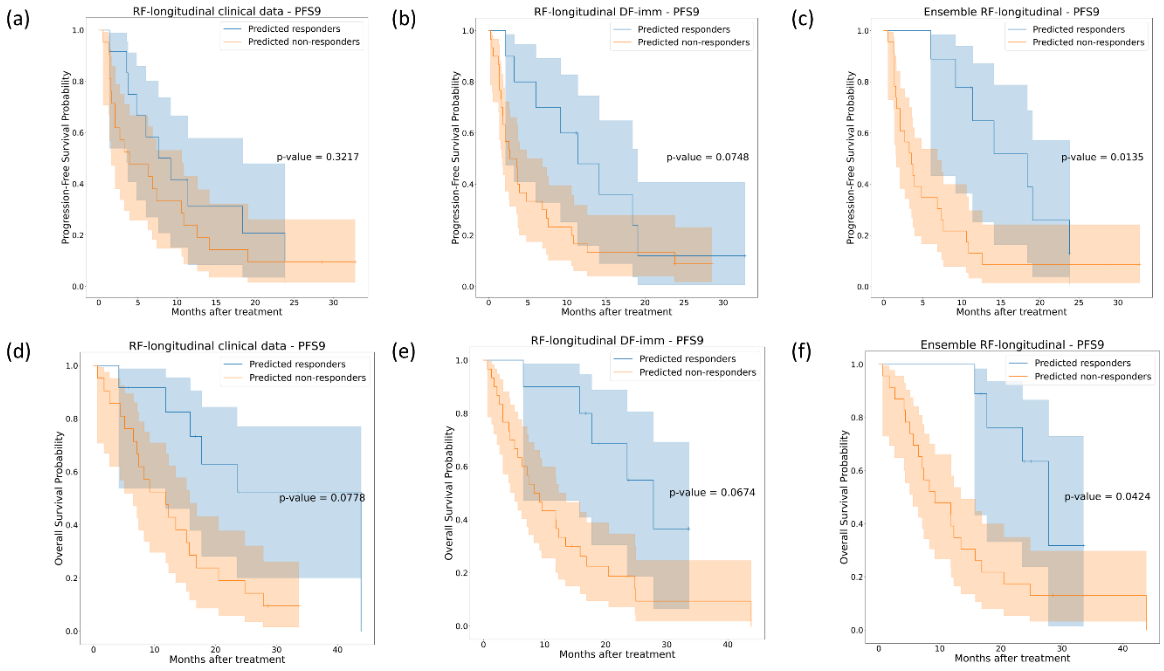

Supplementary Figure S2 Kaplan-Meier survival curves on the independent test cohort for longitudinal RF based on clinical data ((a) and (d)), longitudinal RF with deep features ((b) and (e)) and ensemble RF ((c) and (f)) trained for endpoint PFS9, according to risk groups based on each models' predictions. The first row represents the progression-free survival Kaplan-Meier curves, while the second row represents the overall survival Kaplan-Meier curves.
